# Many dissimilar NusG protein domains switch between α-helix and β-sheet folds

**DOI:** 10.1038/s41467-022-31532-9

**Published:** 2022-07-01

**Authors:** Lauren L. Porter, Allen K. Kim, Swechha Rimal, Loren L. Looger, Ananya Majumdar, Brett D. Mensh, Mary R. Starich, Marie-Paule Strub

**Affiliations:** 1 National Library of Medicine, National Center for Biotechnology Information, National Institutes of Health, Bethesda, MD 20894 USA; 2 National Heart, Lung, and Blood Institute, Biochemistry and Biophysics Center, National Institutes of Health, Bethesda, MD 20892 USA; 3 Howard Hughes Medical Institute, Janelia Research Campus, Ashburn, VA 20147 USA; 4 The Johns Hopkins University Biomolecular NMR Center, The Johns Hopkins University, Baltimore, MD 21218 USA

**Keywords:** Protein structure predictions, Structural biology, Molecular conformation, Protein folding

## Abstract

Folded proteins are assumed to be built upon fixed scaffolds of secondary structure, α-helices and β-sheets. Experimentally determined structures of >58,000 non-redundant proteins support this assumption, though it has recently been challenged by ~100 fold-switching proteins. Though ostensibly rare, these proteins raise the question of how many uncharacterized proteins have shapeshifting–rather than fixed–secondary structures. Here, we use a comparative sequence-based approach to predict fold switching in the universally conserved NusG transcription factor family, one member of which has a 50-residue regulatory subunit experimentally shown to switch between α-helical and β-sheet folds. Our approach predicts that 24% of sequences in this family undergo similar α-helix ⇌ β-sheet transitions. While these predictions cannot be reproduced by other state-of-the-art computational methods, they are confirmed by circular dichroism and nuclear magnetic resonance spectroscopy for 10 out of 10 sequence-diverse variants. This work suggests that fold switching may be a pervasive mechanism of transcriptional regulation in all kingdoms of life.

## Abstract (web summary)

Folded proteins are composed of secondary structures, α-helices and β-sheets, that are generally assumed to be stable. Here, the authors combine computational prediction with experimental validation to show that many sequence-diverse NusG protein domains switch completely from α-helix to β-sheet folds.

## Introduction

For over 60 years, biological science has been heavily influenced by the protein folding paradigm, which asserts that a protein assumes one fold specified by its amino acid sequence^1^. Fold-switching proteins challenge this paradigm by remodeling their secondary and tertiary structures and changing their functions in response to cellular stimuli^2^. These proteins regulate diverse biological processes^3^ and are associated with human diseases such as cancer^4^, malaria^5^, and COVID-19^6^. Nevertheless, the ostensible rarity of fold switching leaves open the question of whether it is a widespread molecular mechanism or a rare exception to the well-established rule.

A major barrier to assessing the natural abundance of fold-switching proteins has been a lack of predictive methods to identify more. Whereas computational methods for rapid and accurate prediction of secondary and tertiary structure for single-fold proteins have been established^7–9^, methods to simply classify fold switchers have lagged. This comparative lack of progress arises from the small number of experimentally observed fold switchers (<100), hampering the discovery of generalizable characteristics that distinguish them from single folders. As a result, essentially all naturally occurring fold switchers have been discovered by chance^10^.

**Fig. 1:**
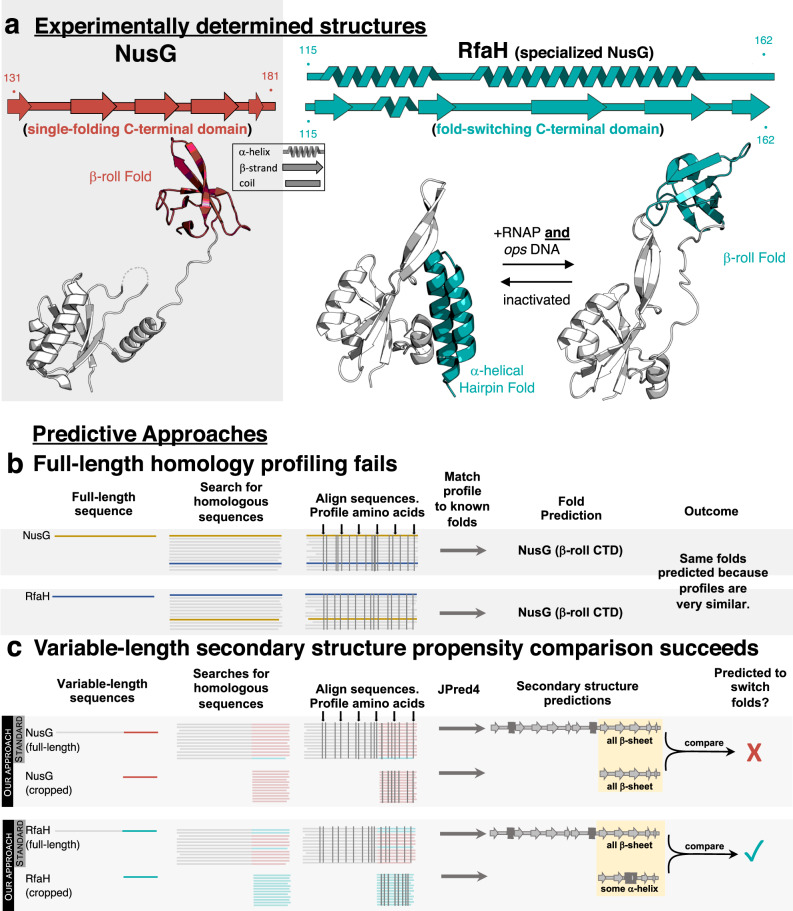
Variable-length secondary structure propensity comparison discriminates between fold-switching RfaH and single-folding NusG. **a** Experimentally determined secondary structures and folds of single-folding NusG (PDB ID: 6ZTJ_CF) and the autoinhibited/active NusG^SP^, RfaH (α-helical hairpin PDB: 5OND_A/β-roll PDB: 6C6S_D, respectively). Dashed lines represent missing density in the NTD of the NusG cryo-EM structure and in the NTD-CTD linker of the RfaH crystal structure. NusG/RfaH CTDs are colored red/teal; NTDs are gray. **b** Profile-based methods fail to identify structural differences between full-length NusG and RfaH because both proteins have similar conservation patterns. Vertical gray bars indicate positions of conserved amino acids. **c** Variable-length secondary structure propensity comparison identifies structural differences between single-folding NusG and fold-switching RfaH. Secondary structure propensities of both the full-length and cropped (CTD) sequences of NusG (above) and RfaH (below) are determined using JPred4. Typically, JPred4 is run on full-length sequences only (“standard” in gray box). While both full-length and cropped NusG sequences have similar amino acid conservation patterns (gray vertical lines, top gray panel), conservation patterns differ for full-length and cropped RfaH (gray vertical lines, bottom gray panel). Similar/different full-length and cropped conservation patterns lead to similar/different secondary structure predictions, suggesting that NusG does not switch folds (top) while RfaH does (bottom). These different patterns likely result from different MSA depths (Fig. S1). Full-length alignments are deeper and have mixtures of both colors, indicating the presence of both fold-switching and single-fold sequences. These mixtures reflect properties of the NusG superfamily. By contrast, cropped sequence MSAs are shallower and homogeneous, reflecting properties of NusG subfamilies. The sequence distributions depicted are for illustrative purposes only since true sequence distributions are unknown. Source data are provided as a Source Data file.

Previously, we developed a sequence-based approach^11,12^ to predict protein fold switching. This approach is based on the observation that the secondary structure of a protein domain or subdomain can change dramatically depending on its context^13–15^. Accordingly, the secondary structure prediction of a fold-switching sequence can change depending on whether it is queried within part of a larger sequence or in isolation^12^. Context-dependent secondary structure is rarely captured by conventional approaches, which tend to predict protein structure using a full amino acid sequence only^16,17^ (Fig. 1b) or subsequences (“crops”) significantly longer than fold-switching regions^18,19^. This problem can be circumvented by comparing secondary structure predictions of whole fold-switching sequences with isolated short (25-40-residues) fragments that could potentially switch folds. Predictions that shift from β-sheet to α-helix—or vice versa—by changing sequence context indicate fold switching with high statistical significance^12^. Secondary structures were predicted using JPred4^20^, a single-hidden-layer neural network trained on 1000 sequence-diverse proteins with solved structures. Previous work showed that JPred4 rarely mistakes α-helices for β-sheets—or vice versa—in single-fold proteins^21^. Furthermore, it predicts fold switching more accurately than other secondary structure predictors because (1) it uses a curated database of non-redundant sequences and (2) it relies primarily on hidden Markov Models (HMMs) rather than position-specific scoring matrices (PSSMs)^11^. HMMs are more sensitive than PSSMs because they assume that insertion and deletion probabilities vary with sequence position and calculate insertion and deletion penalties from input sequence alignments rather than using ad hoc parameters^22^. For instance, this sensitivity allowed JPred4 to predict dramatic changes in secondary structure resulting from a single amino acid substitution^11^.

Fold-switching has been shown to occur in the NusG protein superfamily, which comprises both single-fold and fold-switching proteins. NusGs are the only family of transcriptional regulators known to be conserved from bacteria to humans^23^. Housekeeping NusGs (hereafter called NusGs) exist in nearly every known bacterial genome and associate with transcribing RNA polymerase (RNAP) at essentially every operon, where they often promote transcription elongation by reducing RNAP pausing. NusG homologs from other kingdoms of life, such as DSIF in humans and Spt5 in archaea and yeast, are also called NusGs in this paper. Specialized NusGs (NusG^SP^s), which also exist in all kingdoms of life, promote transcription elongation at specific operons only^24^. Furthermore, some NusGs and NusG^SP^s couple transcription with other biological processes such as translation^25^, RNA silencing^26^, and chromatin modification^27^. Atomic-level structures of NusGs from several organisms have been determined^28–32^. Bacterial NusGs share a two-domain architecture with a NusG N-terminal (NGN) domain that binds RNAP, and a C-terminal Kyprides, Ouzounis, Woese (KOW) domain, which assumes a β-roll fold. The structure of *Escherichia coli* NusG is shown in Fig. 1a. The only NusG^SP^ with an experimentally determined full-length structure^25,33^ is *E. coli* RfaH (Fig. 1a), whose sequence is 19% identical and 37% similar to that of *E. coli* NusG. While the N-terminal domain of *E. coli* RfaH maintains the NGN fold and RNAP-binding activity of its housekeeping NusG homologs, its C-terminal domain (CTD) switches between two disparate folds: an α-helical hairpin that inhibits RNAP binding except at operon polarity suppressor (*ops*) DNA sites and a β-roll that binds the S10 ribosomal subunit, fostering efficient translation^25^ (Fig. 1a). This reversible change in structure and function is triggered by binding to both *ops* DNA and RNAP^34^. Thus, RfaH’s fold-switching CTD serves two purposes: (1) to regulate the N-terminal domain (NTD) so that it associates with RNAP exclusively at *ops* sites and (2) to foster efficient translation of transcripts produced by RNAP when bound to its NTD.

In this work, we focus on bacterial two-domain NusGs and hypothesize that the CTDs of fold-switching NusGs, such as RfaH, are predisposed to fold into both α-helical and β-sheet structures while single-fold NusGs are predisposed to fold into β-sheets only. Accordingly, RfaH’s CTD folds into an α-helical hairpin when expressed with its N-terminal NGN domain but into a β-roll when expressed in isolation^25^. Thus, our approach compares the predicted secondary structures of both the full-length amino acid sequence (N-terminal NGN domain+CTD) and the isolated (cropped) C-terminal domain. CTDs with predicted β-sheet secondary structures in both full-length and cropped sequences are expected to be single folders with constant β-sheet propensities. By contrast, CTDs with regions whose predicted secondary structures change from β-sheet to α-helix when their sequences change from full-length to cropped are expected to switch folds (Fig. 1c). Applying this approach to all ~15,000 sequences in the NusG superfamily, our approach predicted that 24% of NusG-like sequences switch between α-helix and β-sheet folds, a proportion significantly larger than the 0.5-4% predicted previously^2^.

## Results

### Pervasive fold switching predicted in the NusG superfamily

Our approach was tested on 15,516 nonredundant NusG/NusG^SP^ sequences (Methods, Supplementary Data 1). Consistent with other methods (Fig. 1b), it predicted that 95% of CTDs would assume β-sheet folds when full-length sequences were used as inputs. By contrast, 24% of cropped CTD sequences (>3600) were predicted to have substantial α-helical content (Methods), suggesting that they switch folds. These prediction differences likely arise from the multiple sequence alignments (MSAs) used to generate predictions (Fig. 1c). N_eff_ values, which quantify MSA depth and diversity^35^, were ~3800 larger, on average, for PSI-BLAST^36^-generated MSAs from full-length input sequences than from their cropped counterparts (Fig. S1, Methods). Thus, full-length alignments tend to be >3X deeper than cropped. As evidenced by the higher level of predicted β-sheet, these deeper, more diverse alignments seem to reflect properties of the NusG superfamily, whose CTDs can presumably fold into β-roll structures regardless of whether they switch folds. Conversely, shallower CTD alignments seem to reflect properties of NusG subfamilies, some of whose members have CTDs with helical propensities, such as *E. coli* RfaH, while others maintain β-sheet propensities, such as *E. coli* NusG.

To estimate the false-negative and false-positive rates of these predictions, we exploited known operon structures of NusG and several specialized homologs^24^ as an orthogonal method to annotate sequences as NusGs or NusG^SP^s. We mapped the sequences used for prediction to solved bacterial genomes (Methods) and analyzed each sequence’s local genomic environment for signatures of co-regulated genes. Of the 15,195 total sequences, 5175 mapped to contexts consistent with housekeeping NusG function. Only 26 of these were predicted to switch folds, suggesting a false-positive rate of 0.5% for fold-switch predictions. Performing a similar calculation in 849 previously identified RfaHs^24^ (Supplementary Data 1), 31 were predicted single folders. These results suggest that fold switching is widely conserved among RfaHs, which, if correct, indicates a false positive rate of 4% (31/849). Full-length *Vibrio cholerae* RfaH, whose sequence is 44% identical to *E. coli* RfaH, was characterized by NMR and found to have a helical CTD in a recent preprint^37^. This result further indicates that fold switching is conserved among RfaHs. Of the remaining 8661 sequences with high-confidence predictions (Methods) – encompassing several NusG^SP^ clades –31% were predicted to switch folds.

### Experimental validation of fold-switch predictions

**Fig. 2:**
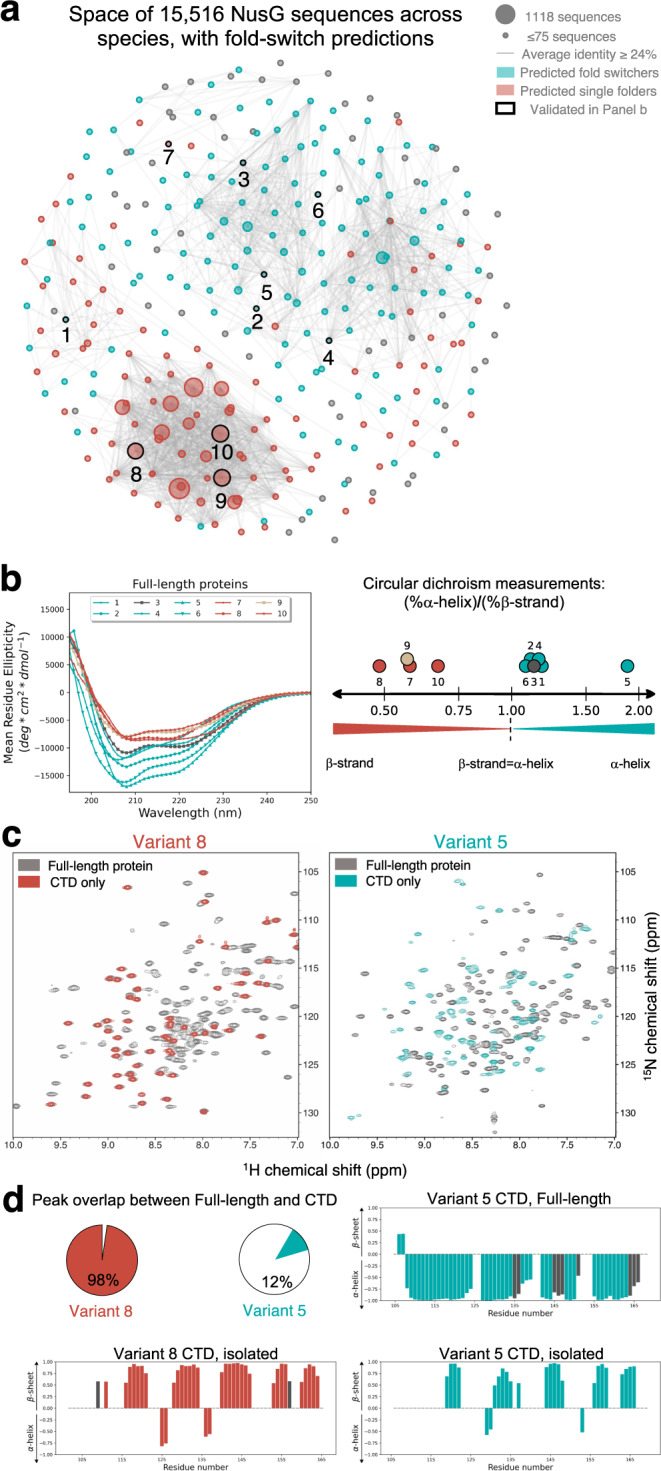
RfaH/NusG sequence space. **a** Force-directed graph of 15,516 full-length RfaH/NusG sequences. The largest node contains 1118 sequences; all nodes with 75 sequences or fewer are the same (smallest) size. Edges connecting the graph represent an average aligned identity between the sequences in two nodes ≥24%. Nodes labeled in teal/red were predicted to be fold switchers/single folders, on average; gray nodes contained only sequences with low-confidence predictions. Nodes with successfully purified and characterized variants are outlined in black; nodes with all experimentally tested variants are shown in Fig. S3. **b** CD spectra of all full-length constructs cluster into RfaH-like (teal) and NusG-like spectra (red). Fractions of α-helix:β-sheet measured from these spectra are shown to their right. All ratios for predicted fold switchers are larger than 1.0; all ratios for predicted single folders are less than 0.75. Numerical labels shown in (**a**) correspond to variant numbers. Numbers are shown on a log_2_ scale. *E. coli* RfaH (Variant #3) and *E. coli* NusG (Variant #9) references are colored gray and beige, respectively, in both panels. **c** The ^1^H-^15^N HSQCs of full-length and CTD variants of a putative single-folder (Variant #8) are nearly superimposable, By contrast, the HSQCs of full-length and CTD variants of a putative fold switcher (Variant #5) differ significantly. **d** Percentages of HSQC overlap from (**c**) are quantified: 98% overlap for Variant 8 demonstrates that its CTD does not switch folds. Its isolated CTD was assigned and found to assume β-sheet secondary structure (bottom right). By contrast, 12% overlap for Variant 5 suggests that its CTD might switch folds. Its CTD was assigned by NMR in both full-length (top right) and isolated (bottom right) forms. Consistent with fold switching, its CTD in the full-length form was found to be α-helical, while its isolated CTD was found to be β-sheet. Colored bars are based on chemical shift assignments; gray non-zero bars show secondary structure predictions based on computational modeling only (Methods). Source data are provided as a Source Data file.

A representative group of variants with dissimilar sequences was selected for experimental validation. First, all NusG-superfamily sequences were clustered and plotted on a force-directed graph, hereafter called NusG sequence space (Fig. 2a, Supplementary Fig. 2, Supplementary Data 1). The map of this space, in which clusters with higher sequence similarity are grouped closer in space, revealed that some putative fold-switching/single-folding nodes cluster together within sequence space (upper/lower groups of interconnected nodes), while other regions had mixed predictions (left/right groups of interconnected nodes). Sixteen candidates selected for experimental validation came from distinct nodes, had diverse genomic annotations, and originated from different bacterial phyla (Supplementary Fig. 3, Supplementary Tables 2, 3). Of these 16 candidates, 10 could be expressed and purified (Supplementary Table 1).

Circular dichroism (CD) spectra of 10 full-length variants were collected. We expected the spectra of fold switchers to have more helical content than single folders because their CTDs have completely different structures (RfaH: all α-helix ground state, NusG: all β-sheet ground state), while the secondary structure compositions of their single-folding NTDs are expected to be essentially identical. *E. coli* RfaH (variant #3) and *E. coli* NusG (variant #9) were initially compared because their atomic-level structures have been determined (Fig. 1a)^38,39^. As expected, their CD spectra were quite different (Supplementary Fig. 4a): *E. coli* RfaH had a substantially higher α-helix:β-strand ratio (1.1) than *E. coli* NusG (0.57) – consistent with solved structures (Fig. 2b, variants #3 and #9).

All remaining predictions were also consistent with the CD spectra of their corresponding variants (Fig. 2b, Supplementary Table 2). Specifically, five predicted fold switchers had RfaH-like CD spectra with minima at 208 nm, a characteristic feature of helical folds that suggests ground-state helical bundle conformations in two RfaHs (variants #2, #6), a LoaP (variant #1), an annotated NusG (variant #4), and an annotated “NGN domain-containing protein” (variant #5). Interestingly, all five of these variants had essentially as much predicted helical content as the reference fold switcher, *E. coli* RfaH (α-helix:β-strand ratio ≥1.1), further suggesting ground-state helical CTDs. Additionally, the remaining three predicted single folders had NusG-like CD spectra that lacked minima at 208 nm: two annotated NusGs (variants #8, #10) and one UpbY/UpxY (variant #7).

We then assessed whether putative fold-switching CTDs could assume β-sheet folds in addition to the α-helical conformations suggested by CD. Previous work^25^ has shown that the full-length RfaH CTD folds into an α-helical hairpin while its isolated CTD folds into a stable β-roll. Thus, we determined the CD spectra of nine isolated CTDs: six from putative fold switchers and three from putative single folders; the tenth (Variant 7 CTD) was degraded during expression on two independent occasions. All spectra had low helical content and high β-sheet content (Supplementary Fig. 4b), suggesting that the CTDs of all six predicted fold switchers can assume α-helical hairpin folds in their full-length forms and β-roll folds when expressed in isolation.

CD can potentially mislead since it shows aggregate, rather than residue-specific, protein properties. Thus, it is possible that the higher helical content observed in Variants 1-6 could result from their NTDs rather than their CTDs. Though unlikely, since the NGN fold of the NTD is conserved from bacteria to humans^23^, we investigated this possibility for two variants at higher resolution using nuclear magnetic resonance (NMR) spectroscopy – which assigns residue-specific structure. Previous work^25^ has shown that the isolated CTD of RfaH, which folds into a β-roll, has a significantly different 2D ^1^H-^15^N Heteronuclear Single Quantum Coherence (HSQC) spectrum than full-length RfaH, whose CTD folds into an α-helical hairpin. Thus, we conducted similar experiments on one single-folding variant (#8) and one putative fold switcher (Variant #5). The backbone amide resonances of the full-length and CTD forms of Variant #8 were 98% superimposable, whereas only 12% of peaks from the full-length and CTD forms of Variant #5 overlapped (Fig. 2d). This result demonstrates that, as predicted, Variant #8 does not switch folds. It is also consistent with the prediction that Variant #5 switches folds because large backbone amide shifts can suggest a fold switch, though large shifts can also result from changes in CTD:NTD interactions without a significant conformational shift^29^. Subsequently, assigned backbone amide resonances were used to characterize the secondary structures of Variant CTDs at higher resolution using TALOS-N^40^ (Methods, Fig. 2d, Supplementary Fig. 4c, Supplementary Table 3). Both isolated CTDs had secondary structures consistent with the β-roll fold. Combining this result with the 98% peak overlap between full-length Variant #8 and its CTD (Fig. 2d) indicates that Variant #8’s CTD maintains a β-roll fold. Alternatively, the TALOS-N secondary structure predictions calculated from chemical shift assignments of the full-length Variant #5 CTD indicate that it is largely helical (Fig. 2d), demonstrating that it switches folds.

These results, though covering a very small proportion of the sequences in this superfamily, support the accuracy of our predictions and indicate that:

1. Some, but not all, NusG^SP^s besides RfaH probably switch folds. Specifically, full-length LoaP (variant #1), which regulates the expression of antibiotic gene clusters^41^, had an RfaH-like CD spectrum, whereas full-length UpbY, a capsular polysaccharide transcription antiterminator from *Bacillus fragilis* (variant #7), appears to assume a NusG-like fold.
2. Some annotated NusGs have RfaH-like CD spectra (variant #4), likely the result of incorrect annotation. Indeed, the genomic environment of variant #4 (Methods) suggests that is a UpxY, not a NusG.
3. The fold-switching mechanism appears to be conserved among annotated RfaHs with low sequence identity (≤32%, variants #2, #3, and #6), a possibility proposed previously^42^, though without experimental validation. Also, “NGN domain-containing protein” variant #5 is genomically inconsistent with being a NusG and is likely an RfaH.

### Other predictive methods do not capture the helical ground state of fold-switching variants

To benchmark the performance of our secondary-structure-based approach, we assessed whether machine learning and template-based methods could also distinguish between fold switchers and single folders in the NusG superfamily. Specifically, we tested AlphaFold2^8^, Robetta^43^, EVCouplings^16^, and Phyre2^17^ on variants #1-6, whose CD spectra were all RfaH-like, suggesting that their CTDs assume ground-state helical folds. All methods predicted only one CTD conformation per variant – almost all of which were β-sheet (Supplementary Fig. 5), except for AlphaFold2’s predictions of *E. coli* RfaH (variant #3), whose experimentally determined structure^38^ was in its training set, and variant #6, whose sequence is nearest and best connected with *E. coli* RfaH in Sequence Space (Fig. 2a). Predicted amino acid contacts from Robetta and EVCouplings corresponded with the NusG-like β-roll fold for *E. coli* RfaH (Supplementary Fig. 6). The MSAs used for these alignments were deep: N_eff_ of 9633 and 17,245 for Robetta and EVCouplings, respectively. As shown with the alignments used for JPred4 predictions (Supplementary Fig. 1), these deep sequence alignments might capture folding properties of the NusG superfamily rather than the RfaH subfamily.

**Fig. 3:**
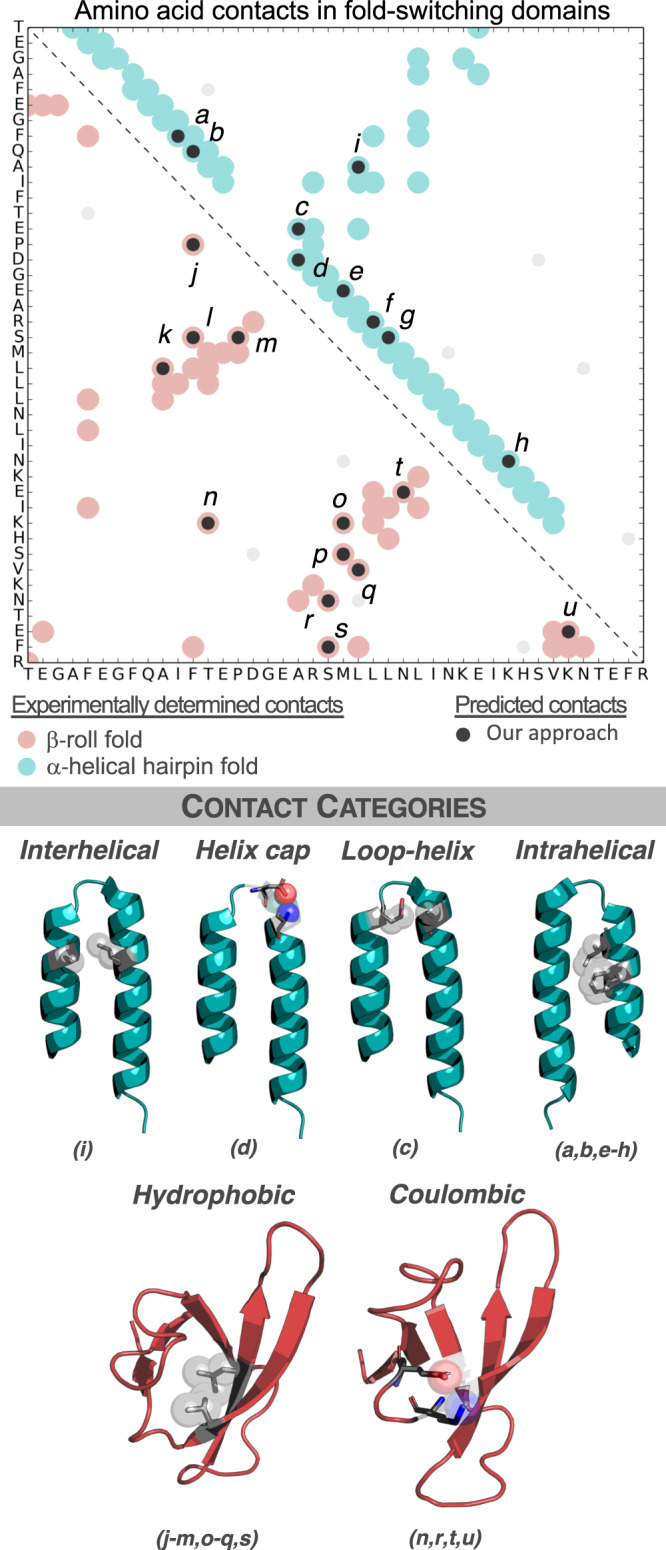
Fold-switching sequences have conserved amino acid contacts from both folds. Predicted amino acid contacts from fold-switching sequences (dark gray circles) correspond to both the β-roll fold (PDB ID: 2LCL, red circles) and the α-helical hairpin fold (PDB ID: 5OND, chain A, teal circles). Couplings that do not correspond to experimentally observed contacts are shown as light circles. Categories of amino acid contacts from both folds use the alphabetically labeled contacts in the plot above them. Source data are provided as a Source Data file.

Coevolutionary analysis was performed on a subset of sequences that our approach predicted to switch folds (Methods). Specifically, we clustered putative fold switchers by their secondary structure predictions and did coevolutionary analysis on the cluster containing the *E. coli* RfaH sequence using GREMLIN^44^. The residue-residue contacts generated from these sequences differed substantially from the NusG-like couplings generated before (Fig. 3). Furthermore, GREMLIN couplings calculated from the alignments used by EVCouplings and Robetta corresponded with the β-roll fold only (Supplementary Fig. 6), demonstrating that the JPred-filtered sequence alignment—not the GREMLIN algorithm—was responsible for the discovery of alternative contacts. These results again indicate that shallower alignments—such as the JPred-filtered one (N_eff_ = 834) —reflect folding properties of the RfaH subfamily while deeper alignments (N_eff_ = 9633 and 17,245 for Robetta and EVCouplings, respectively), reflect folding properties of the NusG superfamily, whose members largely do not switch folds.

This analysis of putative fold-switching CTDs indicates evolutionary coupling of residue-residue contacts unique to two distinct folds. For the α-helical fold, six intrahelical hydrophobic contacts and one set each of interhelical contacts, strand-helix contacts, and helix-capping contacts were observed (Fig. 3). Overall, 96% of interhelical contacts were hydrophobic, 94% of helix-capping residues could potentially form an i-4→i or i→i backbone-to-sidechain hydrogen bond, 85% of residues in the helix-loop interaction had a charged residue in one position (but not both), and 80% of residues in intrahelical contact *a* were both hydrophobic. The remaining contacts gave more mixed results, perhaps due to hydrophobic residues contacting the hydrophobic portion of their hydrophilic partners. Contacts from the β-roll fold, identified by both GREMLIN and EVCouplings/Robetta, were categorized as Coulombic and hydrophobic (Supplementary Fig. 7). Previous work has shown that interdomain interactions also contribute significantly to RfaH fold switching^25^. Unfortunately, these interactions could not be identified by coevolutionary analysis (Supplementary Fig. 8), a likely result of the limited number of JPred-filtered sequences available.

### Fold-switching CTDs are diverse in sequence, function, and taxonomy

**Fig. 4:**
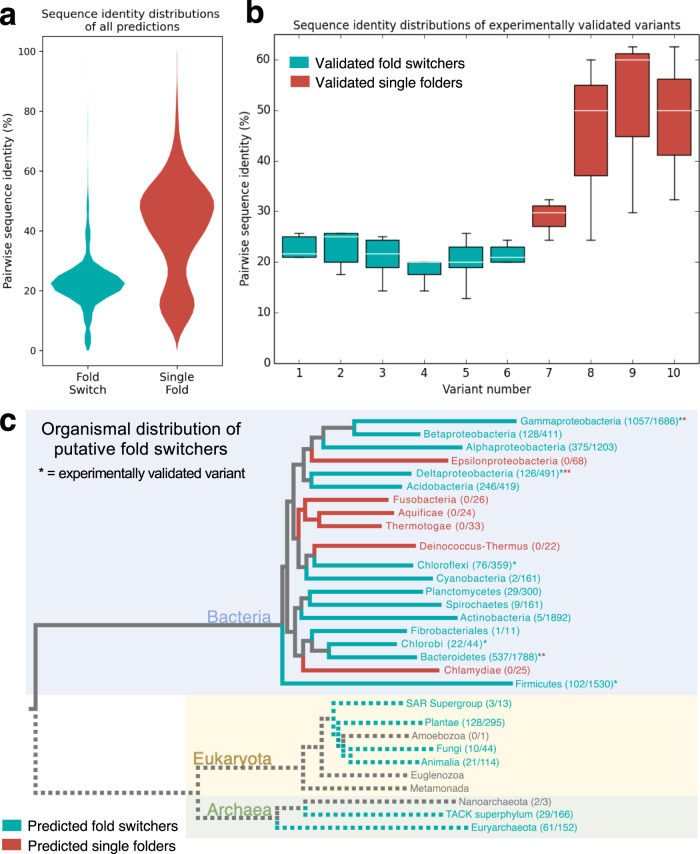
The sequences of fold-switching CTDs are highly diverse and found in a wide variety of bacterial phyla. **a** Violin plots of pairwise sequence identities differ significantly for putative fold switchers and putative single folders. On average, pairwise sequence identities are lower for putative fold switchers (teal, 20.4%) than single folders (red, 40.5%). **b** Box-and-whiskers plots of pairwise sequence identities of fold-switching and single-folding CTDs of variants 1-10 in Fig. 2. The distributions of each teal (fold-switching)/red (single-fold) box were derived from n = 5/3 independent pairwise identities; each box bounds the interquartile range (IQR) of the data (first quartile, Q1 through third quartile, Q3); medians of each distribution are shown in white; lower whisker is the lowest datum above Q1-1.5*IQR; upper whisker is the highest datum below Q3 + 1.5*IQR. These distributions are consistent with the violin plots in panel (**a**). **c** Fold-switching CTDs are predicted in many bacterial phyla (blue background) and other kingdoms of life. Numbers next to taxa represent #predicted fold switchers/#total sequences. Gray branches represent unidentified common ancestors, since the evolution of fold-switching NusGs is unknown. Dotted lines represent lower-confidence predictions since fold switching has not been confirmed experimentally in Archaea (green background) and Eukaryota (yellow background). Fold-switching/single-folding predictions are represented by teal/red colorings; predictions in branches with fewer than 10 sequences are gray. Source data are provided as a Source Data file.

It might be reasonable to expect fold-switching CTD sequences to be relatively homogeneous, especially since variants of another fold switcher, human XCL1, lose their ability to switch folds below a relatively high identity threshold (60%)^45^. The opposite is true. Sequences of putative fold-switching CTDs are substantially more heterogeneous (20.4% mean/19.4% median sequence identity) than sequences of predicted single folders (40.5% mean/42.5% median sequence identity, Fig. 4a). Accordingly, among the sequences tested experimentally, similar mean/median sequence identities were observed: 21.0%/21.1% (fold switchers), 43.2%/41.2% (single folders, Fig. 4b). Furthermore, fold-switching CTDs were predicted in most bacterial phyla, and many were predicted in archaea and eukaryotes as well (Fig. 4c, Supplementary Data 2). These results suggest that many highly diverse CTD sequences can switch folds between an α-helical hairpin and a β-roll in organisms from all kingdoms of life.

## Discussion

Why might the sequence diversity of fold-switching CTDs exceed that of single folders? Functional diversity is one likely explanation^46^. Previous work has shown that NusG^SP^s drive the expression of diverse molecules from antibiotics to toxins^24^. Our approach suggests that many of these switch folds. Furthermore, since helical contacts are conserved among at least some fold-switching CTDs, it may be possible that CTD sequence variation is less constrained in other function-specific positions. The fold-switching mechanism of RfaH allows it to both regulate transcription and expedite translation, presumably quickening the activation of downstream genes. Fold-switching NusG^SP^s are likely under strong selective pressure to conserve this mechanism when the regulated products control life-or-death events, such as the appearance of rival microbes or imminent desiccation. Supporting this possibility, NusG^SP^s usually drive operons controlling rapid response to changing environmental conditions such as macrolide antibiotic production^41^, antibiotic-resistance gene expression^24^, virulence activation^47^, and biofilm formation^48^.

Our approach was sensitive enough to predict fold-switching proteins, setting it apart from other state-of-the-art methods. These other methods assume that all homologous sequences adopt the same fold, as evidenced by their use of sequence alignments containing both fold-switching and single-folding sequences. These mixed sequence alignments biased their predictions. While those predictions are partially true since both fold-switching and single-folding CTDs can fold into β-rolls, they miss the alternative helical hairpin conformation and its regulatory function^31^. Computational approaches that account for conformational variability and dynamics, a weakness in even the best predictors of protein structure^8^, could lead to improved predictions. This need is especially acute in light of recent work showing how protein structure is influenced by the cellular environment^49^, and it could inform better design of fold switchers, a field that has seen limited success^50–52^.

Our results indicate that fold switching is a pervasive, evolutionarily conserved mechanism. Specifically, we predicted that 24% of the sequences within a ubiquitous protein family switch folds and observed coevolution of residue-residue contacts unique to both folds. This sequence-diverse dual-fold conservation challenges the protein folding paradigm and indicates that foundational principles of protein structure prediction may need to be revisited.

This work has two major limitations. Firstly, the level of error in our predictions is unknown. Due to the limited number of known fold-switching proteins, robust error rates of JPred4 as a fold-switch predictor cannot be determined. Although our experimental results suggest that the approach is accurate in all ten cases tested, it is uncertain how well it performs in the full NusG superfamily (~15,000 proteins) or on proteins in general. Our orthogonal computational analysis indicates that the predictions capture single-folding NusGs 99.5% percent of the time. It is less clear how accurately they capture fold switching in NusG^SP^s, since some do not switch folds (e.g., Variant #7) and others do (Variants 1–6). Thus, additional work is needed to assess error rates and sources of error from this approach. Secondly, CD does not provide residue-specific structural information. Thus, it is possible that helical character arising outside of NusG CTDs could lead to the RfaH-like CD spectra observed in Variants 1-6. This possibility seems unlikely, however, given that all 6 variants are two-domain proteins whose N-terminal NGN domains are highly conserved^23^. Furthermore, Variants #3 and #5 have been shown by NMR previously^25^ or here to assume two folds.

The success of our method in the NusG superfamily suggests that it may have enough predictive power to identify fold switching in protein families where only single folders have been observed to date. Such predictions would be particularly useful since many fold switchers are associated with human disease^3–6^. Given the unexpected abundance of fold switching in the NusG superfamily, there may be many more unrelated fold switchers to discover.

## Methods

### Identification of NusG-like sequences

NusG-like sequences were identified from the October 2019 Uniprot90 database^53^ using an iterative BLAST^36^ approach. Specifically, the *E. coli* RfaH sequence (Uniprot ID Q0TAL4) was BLASTed against the database. All hits with a maximum e-value of 10^−4^ were aligned using Clustal Omega^54^, which generated their sequence identity matrices from the resulting alignment. Sequences were clustered by their identities using the agglomerative clustering algorithm from the python module scikit-learn^55^. Sequence identity between proteins in each cluster was ≥78%. Randomly selected sequences from the 25 largest clusters were then individually BLASTed against the Uniprot90 database, and the resulting hits were combined; redundant identical hits from independent searches were removed. This procedure (search-align-cluster) was repeated two additional times to generate the full list of 15,516 sequences in 305 clusters.

### Determination of CTDs

Sequences of annotated RfaHs were aligned to the sequence of *E. coli* RfaH (Uniprot ID Q0TAL4) using Clustal Omega^54^. CTDs were defined as up to 50 residues, but not shorter than 40 if the CTD region comprised <50 residues, beginning with the positions that aligned to the RfaH sequence *KVIIT*. Sequences of proteins not annotated as RfaH were aligned to the *E. coli* NusG sequence (Uniprot ID P0AFG0) using Clustal Omega. CTDs were defined as 50 residues beginning with positions that aligned the NusG sequence *EMVRV*. Because of their diversity, sequences from each individual cluster were aligned against the NusG sequence separately, each using Clustal Omega. The number of sequences with CTDs long enough to make these predictions totaled 15,195 (Supplementary Data 1), 98% of all NusG-like sequences identified.

### JPred4 predictions

JPred4^20^ predictions were carried out as in^11^, as follows. PSI-BLAST^36^ searches were run all 50-residue CTD sequences using two databases: the JPred database (http://www.compbio.dundee.ac.uk/jpred/about_RETR_JNetv231_details.shtml) from 2014 and the Uniprot90 database from January 2021. The resulting sequences were aligned with MView^56^ and inputted into HMMer^57^ 2.3.2 to generate a Hidden Markov Model (HMM). The resulting HMM was converted to GCG using hmmconvert and converted to JPred4 input using the activation function:

1 $$\frac{1}{1+{e}^{-\frac{x}{100}}}$$

The PSI-BLAST-generated position-specific scoring matrix (PSSM) was converted to JPred4 input using the following activation function:

2 $$\frac{1}{1+{e}^{-x}}$$

The converted HMM and PSSMs were inputted into the jnet 2.3.1 algorithm^58^, and jnetpred predictions were used to assess fold switching.

Sequences of each prediction were aligned against the *E. coli* NusG sequence (beginning with *EMVRV*) using Biopython^59^ Bio.pairwise2.localxs with gap opening/extension scores of −1.0/−0.5. Secondary structure predictions of the sequence in question and of *E. coli* NusG were reregistered according to the resulting pairwise alignments and compared as in^11^. Predictions for *E. coli* NusG were ---EEEEEEEE----EEEEEEEE------EEEEEEEEE—for JPred4 database and ---EEEEEEE-----EEEEEEEEE-----EEEEE------ for UniRef90 database, where – is predicted coil and E is predicted β-sheet. This resulted in a consistent reference for all CTD predictions made. As in^11^, helix to strand discrepancies ≥5% were considered to indicate fold switching. Predictions were considered high-confidence if at least 5 sequences were in the MView^56^-generated alignments used by JPred4. Importantly, JPred4’s training set includes one NusG (PDB ID: 1m1h_A) but excludes RfaH.

We found that the first 10 residues in these 50-residue sequences were similar enough to NusG CTDs that NusG-like sequences overwhelmed sequence alignments informing the predictions, and many likely fold-switching sequences were predicted to be single folders. To circumvent this problem, predictions from both databases were rerun on 40-residue sequences (starting with the first residue that aligned to *ADFNG…* for NusG sequences and *FQAIF*… for RfaH sequences). Predictions were made as with 50-residue sequences. All predictions reported in the main text were from 40-residue sequences, except those in Fig. 1b.

### MSA depths

MSA depths were determined using N_eff_, the effective number of non-redundant sequences in an alignment^35^. We calculated N_eff_ by running GREMLIN^44,60^ on the PSI-BLAST-generated sequence alignments used for JPred predictions using a maximum sequence identity of 90%. Depth differences were computed by subtracting the N_eff_ of a CTD’s MSA from the N_eff_ of its corresponding full-length sequence (Supplementary Fig. 1).

### Force-directed graph

The 305 clusters generated from all full-length NusG sequences were plotted on a force-directed graph using the *spring_layout* function from python NetworkX^61^ with a spring constant of 0.3 and 1000 iterations. Nodes with ≥50% of sequences predicted to switch folds were colored teal; nodes with <50% of sequences predicted to switch folds were colored red. Nodes with no predictions were colored gray. Nodes 1 and 7 were colored differently from their average predictions (single folding, Node 1; fold-switching, Node 7) to highlight the prediction of the sequence validated experimentally, which differed from the average. Edges represented average pairwise identities between nodes ≥24%, a threshold taken from^62^ for sequences of 162 residues (the length of *E. coli* RfaH).

### Genomic analysis of sequences

The annotated genomes (protein.fasta and.gtf annotation) of 31,554 bacterial species were downloaded from Ensembl Bacteria in April 2021. Genomic annotation of NusG was defined as being within 10 kb of a gene annotated as either “SecE,” “RplK,” “RplA,” or “ribosomal protein L11” by text matching. Most bacterial genomes are incompletely assembled and annotated – the genes were required to be within the same chromosome, contig, or plasmid. Each Uniprot sequence in the database of 15,516 was mapped to an Ensembl locus if the species was consistent, and if sequence identity was greater than 90%. Annotation was fetched from Ensembl, as well – this was usually, but not always, consistent with the Uniprot annotation.

Of the 15,516 Uniprot sequences, 7975 mapped to Ensembl genomes. Cursory analysis of some non-mapping sequences suggested that: 1) some Ensembl genomes had incomplete collation of all ORFs, and 2) there were frame shifts and other errors in some Uniprot sequences and some Ensembl genomes. This was also the case for some of the sequences predicted to potentially be fold-switching NusGs: for instance, Uniprot entry A0A0T8ANM4 is frame-shifted relative to the Ensembl genome, producing a C-terminal sequence predicted to switch folds.

Of the 5,435 sequences that mapped to Ensembl loci with *SecE*/*RplK*/*RplA* within 10 kb, only 22 had a separation of >1 kb, and only 59 had a separation of >270 bp – this set of 59 includes 4 proteins predicted to be fold-switching, one of which is a verified RfaH from^24^, indicating that a shorter threshold of distance to *SecE*/*RplK*/*RplA*, perhaps coupled with determining distances from several other conserved *NusG*-*SecE* operon genes, could reduce the false-positive rate caused by mistakenly annotating NusG^SP^s as housekeeping NusGs.

For a small number of sequences that mapped to qualitatively dissimilar genes (e.g., one genomically consistent as being a NusG, another not), the 2nd mapping is given in Data S1, beginning in column AH.

Additionally, of the 600 RfaH sequences that mapped to an annotated Ensembl locus, only one fell within a NusG-like operon (~7 kb away).

### Expression and purification of variants 1-16

Genes encoding all variants were ordered from IDT as gBlocks; all were codon optimized for *E. coli*. Except for the gene encoding variant #7, these genes were digested with HindIII and EcoRI and incorporated into the pPAL7 vector (Bio-Rad) with an N-terminal 6-His tag cloned using a Q5 mutagenesis kit (New England Biolabs); we call this modified vector hispPAL7. In further detail, hispPAL7 was also digested with HindIII and EcoRI. Digested plasmid and digested genes were individually purified with a QIAquick PCR Purification kit (Qiagen). Their concentrations were measured at an absorbance of 260 nm with a NanoDrop One (Thermo Scientific). Digested and cleaned plasmid was combined with each digested and cleaned gene individually at a 1:5 plasmid:gene molar ratio. Each plasmid-gene combination was ligated with 1 μL T7 DNA Ligase and T7 DNA Ligase buffer (New England Biolabs) diluted to 1X final concentration. Reactions were incubated at room temperature for 1 hour, and 5 μL of each reaction was transformed directly into *E. coli* DH5-α cells (New England Biolabs), and plated on Luria Broth (LB) agar plates with 100 μg/mL ampicillin overnight. Two colonies from each plate were picked individually and grown overnight in 3 mL LB with 100 μg/mL ampicillin at 37 °C, shaking at 225 RPM. Plasmids were purified using the Qiaprep Spin Miniprep Kit (Qiagen), and the genetic sequences of each variant were confirmed by Sanger sequencing (Psomagen). Plasmids with confirmed genetic sequences were transformed into *E. coli* BL21-DE3 cells (New England Biolabs), grown in LB at 37° to an OD_600_ of 0.6-0.8, after which they were incubated at 20 °C for 30 minutes, induced with 0.1 mM IPTG, and grown overnight, shaking at 225-250 rpm. The gene encoding variant #7 was cloned into the same vector as the other variants using In-Fusion and expressed as the other variants but at 18 °C instead of 20 °C. The cells from all cultures were pelleted at 10,000*xg* for 10 minutes at 4 °C, resuspended in 2 mL lysis buffer (50 mM Tris, 150 mM NaCl, 5% glycerol, 1 mM DTT, 10 mM imidazole, pH 8.7) and frozen at −80 °C for later purification. Sequencing of all variants was verified by Psomagen.

Thawed cell pellets were resuspended in 25 mL lysis buffer per 1 L of culture grown. 100 mg of DNAseI, 5 mM CaCl_2_, 5 mM MgSO_4_ and 1/2 of a cOmplete EDTA-free protease cocktail inhibitor tablet (Roche) were added per 25 mL of lysis buffer. Cells were lysed by 2 passes through an EmulsiFlex-C3 homogenizer (Avestin). The homogenized lysate was centrifuged for 45 minutes at 40,000*xg* at 4 °C, and its soluble fraction was loaded immediately onto either a 1 mL Ni column (GE HisTrap HP) or an Econo-Pac (Bio-Rad) gravity column with 0.5-1 mL IMAC Ni Resin (Bio-Rad). Soluble lysate was stored on ice while loading at 1 mL/minute through the sample pump of a room-temperature ÄKTA Avant onto a 1 mL HisTrap column or gravity columns were loaded and kept at 4 °C. The HPLC Ni columns were washed with 100 mM phosphate and 500 mM NaCl, pH 7.4, equilibrated in 100 mM phosphate, pH 7.4, and eluted by gradient with 0.5 M imidazole, 100 mM phosphate, pH 8.0 at 2 mL/minute on an ÄKTA Avant. The gravity columns were washed and equilibrated with 10 column volumes each of the same buffers, and protein was eluted at 3 different imidazole concentrations: 100 mM, 500 mM and 2 M, all in 100 mM phosphate, pH 7.4-8.0.

Nickel-purified samples were then loaded onto 1- or 5-mL Profinity eXact^63^ columns (Bio-Rad), washed twice with one column-volume of 2 M NaOAc, and eluted with 100 mM phosphate, 10 mM azide, pH 7.4 at 0.2 mL/minute. Cleavage kinetics for some variants (1, 4, and 6) were too slow to get adequate tagless protein. In these cases, columns were equilibrated with 100 mM phosphate, 10 mM azide, pH 7.4 overnight at 4 °C. Tagless protein was concentrated in 10 kDa MWCO concentrators (Millipore), and the buffer was exchanged to 100 mM phosphate, pH 7.4. A small amount of high-molecular-weight impurity (<10% of the sample) from variants #1 and #4 was removed by running the tagless sample through a 50 kDa MWCO concentrator (Millipore) and keeping the low molecular weight fraction that passed through the filter. Sample purities were assessed by gel electrophoresis (Thermo Fisher NuPAGE 4-12% Bis-Tris gels, Thermo Fisher MES buffer, Bulldog Bio Coomassie Stain), and concentrations were measured on a NanoDrop OneC (Thermo Scientific). Homogeneities of full-length variants 1, 2, 4, 5, and 6 were confirmed by Size Exclusion Chromatography (SEC) on a room-temperature ÄKTA Avant at a flow rate of 0.5 mL/min using a Superdex 75 Increase 10/300 column (Cytiva); all were found to be homogeneous and monomeric.

### Variant CTDs

Full-length variants were shortened using Q5 mutagenesis (New England Biolabs; oligonucleotide sequences are in Supplementary Table 4). Their sequences were confirmed by Sanger sequencing (Psomagen) and are reported in Supplementary Table 3. TS or TSW tags were added to most constructs (but not Variant 5 or 8 CTDs) to speed up their cleavage kinetics on the Profinity eXact^63^ column and to improve concentration measurements using absorbance at 280 nm. All variants were expressed and purified as were variants 1–16, using expression temperatures of 20 °C for Variants 2, 5, 8, and 9 and 16° for the rest. We attempted to purify Variant 7 CTD twice, but it showed signs of degradation during expression in both instances.

### Circular dichroism (CD) spectroscopy

CD spectra of all samples were measured within 1-2 days of purification; they were stored at 4 °C until then. All CD spectra were collected on Chirascan spectrometers (Applied Photophysics) in 1 mm quartz cuvettes (Hellma) in 100 mM phosphate, pH 7.4. Protein concentrations ranged from 8 to 12 μM, and scan numbers ranged from 5 to 10, collected at 1 nm/s with a 1 nm step size. Scans were averaged, and averaged baselines of buffer-blank 1 mm cuvettes were subtracted from the spectra. The resulting spectra were converted to units of Molar Residue Ellipticity [*θ*]_MRE_ using Eq. (3):

3 $${\left[\theta \right]}_{{MRE}}=\frac{\theta \times \epsilon }{L\times N\times A}$$

where *θ* is the ellipticity measured by the instrument, $\epsilon$ is the extinction coefficient determined by Expasy Protparam^64^, *L* is the path length of the cuvette, N is the number of amino acids, and *A* is the absorbance measured by a Nanodrop One (Thermo Scientific). Absorbances were measured at 280 nm for all full-length constructs (Supplementary Table 2) as well as the CTDs of Variants 2, 6, 7, and 9, to which a tryptophan was added to the N-terminus (Supplementary Table 3); sequence-based extinction coefficients of these variants were calculated using Expasy Protparam^64^ (https://web.expasy.org/protparam/). Absorbances of Variants 1, 4, 6, and 10 were measured at 205 nm, with extinction coefficients calculated from https://spin.niddk.nih.gov/clore/Software/A205.html^65^. Concentrations $\left(\frac{A}{\epsilon }\right)$ of the CTDs of Variants 3, 5, and 8 were determined using the Bradford Assay against a Bovine Serum Albumin (New England Biolabs) baseline measured with concentrations of 0.1, 0.25, 0.5, 1.0, and 2.0 mg/mL. Concentrations were converted to molarities based on molecular weights calculated using Expasy Protparam^64^. Resulting spectra were entered into the BestSel^66^ webserver (https://bestsel.elte.hu/index.php), so that their ratio of helix (helix+distorted helix):strand (parallel+antiparallel) could be computed. Ratios were calculated for two wavelength ranges (195–250 nm and 200–250 nm) and averaged (Fig. 2b, Source Data).

### Expression and purification of NMR samples

Based on the protocols in^67–69^, *E. coli* BL21 DE3 cells (New England Biolabs) expressing all isotopically labeled samples were grown in LB to an OD_600_ of 0.6 and pelleted at 5000*xg* for 30 minutes at 4 °C. The pellets were resuspended in 1X M9 at half of the initial culture volume and pelleted at 5000*xg* for 30 minutes at 4 °C. Pellets were then resuspended at ¼ initial culture volume in 2X M9, pH 7.0-7.1, 1 mM MgSO_4_, 0.1 mM CaCl_2_, with 1 g ^15^NH_4_Cl/L, and 4 g of either unlabeled or ^13^C-labeled glucose (Cambridge Isotope Laboratory)/L and equilibrated at 20 °C for 30 min, shaking at 225 rpm, then induced with 1 mM IPTG and grown overnight. Cells were pelleted at 10,000× *g* for 10 minutes at 4 °C.

All labeled variants were purified by FPLC (ÄKTA Avant 25) using the same methods as variants 1–10 above in 5 mL HisTrap HP columns (Cytiva) and 5 mL Profinity eXact columns (BioRad).

### ^1^H-^15^N HSQCs of Variants #5 and #8

All spectra were collected on Bruker Avance II 600 MHz spectrometers equipped with z-gradient cryoprobes and processed with NMRPipe^70^. Variant #8 (full-length and CTD) and variant #5 CTD HSQCs were collected in 100 mM phosphate, pH 7.4 with 10% D_2_O added at 298 K. Under those conditions the spectrum of full-length Variant #5 was broad, even with 1 mM DTT added, but peaks narrowed upon changing the buffer conditions to 25 mM HEPES, 50 mM NaCl, 5% deuterated glycerol (Sigma Aldrich), 1 mM DTT, 10% D_2_O, pH 7.5 (hereafter called HEPES buffer), and collecting the spectrum at 308 K. For consistency, a 2D ^1^H-^15^N HSQC of Variant #5 CTD was also collected in HEPES buffer at 308 K, and the superposition of Variant #5 and Variant #5 CTD in HEPES buffer is shown in Fig. 3c. Protein concentrations ranged from 100-300 μM.

### Assignments of Variant #5 CTDs and Variant #8 CTD

^13^C-labeled 5CTD and 8CTD were expressed and purified as above. Buffer used was 100 mM phosphate, pH 7.4 at 298 K (Variant 8 CTD) and 303 K (Variant 5 CTD). Under these conditions, the ^1^H-^15^N HSQC of Variant 5 was essentially the same as that collected in HEPES buffer (Supplementary Fig. 4c). For each variant, HNCACB, CBCA(CO)NH, and HNCO experiments were collected on Bruker Avance II 600 MHz spectrometers with cryoprobes. Spectra of 8CTD (80 μM) were collected using nonuniform sampling and were processed with SMILE^71^. ^2^H,^15^N,^13^C Variant 5 was produced following protocol^68^, except for the expression temperature, which was lowered to 16 °C. A final concentration of 135 μM in HEPES buffer was produced. HNCACB, HNCA, HN(CO)CA, and HNCO experiments were collected on Bruker Avance II 600 MHz spectrometers with cryoprobes. All NMR spectra were processed using NMRpipe^70^. All resonances were assigned manually with NMRfam Sparky^72^, and secondary structures were determined using TALOS-N^40^. We defined coil predictions to have 0 value, while β-sheets and α-helices were assigned positive and negative values, respectively.

### Coevolutionary analysis

Structure predictions of the 6 fold-switching variants were calculated by entering their full-length sequences (Supplementary Data 2) into the EVCouplings^16^, Robetta^43^, and Phyre2^17^ webservers (https://evcouplings.org, https://robetta.bakerlab.org, http://www.sbg.bio.ic.ac.uk/phyre2/html/page.cgi?id=index). EVCouplings predictions with the recommended e-value cutoffs for MSAs for chosen: (Variant 1: e-3, 2: e-5, 3: e-5, 4: e-20, 5: e-5, 6: e-5). High-confidence predictions for shorter sequences of 40 or 50 residues could not be obtained from either EVCouplings or Robetta. Predicted residue-residue contacts of *E. coli* RfaH from EVCouplings/Robetta with probabilities ≥99%/92% were plotted in Supplementary Fig. 6a, b, and residue-residue contacts from GREMLIN^44^ with probabilities ≥90% were plotted in Fig. 3. These thresholds were determined by maximizing the ratio of true positives to false positives. True positives were considered to be couplings with heavy atoms within 5.0 Å in either the 5OND crystal or the 2LCL structures where at least one of the 2 heavy atoms was from a side chain; one additional contact between residues 140 and 151 was added because they were separated by 5.2 Å within the NMR structure and therefore likely within error of 5.0 Å. Contacts were considered hydrophobic if both atoms in contact were hydrophobic, Coulombic if two atoms in contact had opposite charge and C-N-O/C-O-N angles ≥90°, and helix caps if the distance between sidechain donor/acceptor ≤4° and C-N-O/C-O-H angles ≥90°^73^. All distances and angles were calculated using LINUS^74^.

CTD sequences for GREMLIN webserver (http://gremlin.bakerlab.org/submit.php) analysis in Fig. 3 were obtained by clustering all JPred predictions by Affinity Propagation using the python Scikit-learn module^55^ with damping of 0.99 and a maximum number of 10,000 iterations. Affinities were precomputed by comparing each 40-residue prediction position-by-position, with the following scores: identical predictions (EE, HH, --): 0, coil:secondary structure discrepancies (H-, E-, -H, -E): 0.5, and helix:strand discrepancies (HE,EH): 10, and selecting the cluster with the sequence of *E. coli* RfaH (639 sequences). These sequences were aligned with Clustal Omega and inputted into GREMLIN. 4 iterations of HHBlits^75^ were run on the initial alignment with e-values of 10^−10^. Coverage and remove gaps filters were both set to 75.

GREMLIN webserver analyses were run on EVCouplings and Robetta multiple sequence alignments seeded with the sequence of *E. coli* RfaH. These alignments were taken from EVCouplings *align* and Robetta.msa.npz files. No additional iterations of HHPred were run on either alignment. Coverage and remove gaps filters were both set to 75.

### Pairwise sequence identities

Pairwise sequence identity matrices of predicted fold-switching/single-folding CTDs were calculated using Geneious. The alignments for these sequences were first manually curated to remove sequences that did not align well with the majority; manually curated alignments retained at least 98% of all sequences. The mean/median sequence identities of these two groups were determined from the upper triangular portions of each matrix, excluding positions of identity, using numpy^76^. Pairwise sequence identity matrices of the CTDs of the 10 variants were determined with Clustal Omega.

### Phylogenetic tree

The tree in Fig. 4c was generated by downloading the Interactive Tree of Life^77^ (https://itol.embl.de/itol.cgi), loading it into FigTree^78^, and collapsing branches at the phyletic level, except for Proteobacteria, which were left at the class level because of recent phylogenetic work on proteobacterial RfaH^24^.

Bacterial species from each NusG sequence were obtained from their Uniprot headers. These species were mapped to their respective phyla using TaxonKit^79^ and matched with their predictions. Phyla with fold-switching/single-folding predictions were listed using a python script, and branches of the tree were then colored manually in Adobe Illustrator. Experimentally validated variants from two phyla did not show on the dendrogram in Fig. 4: Candidatus Kryptonia and Deferribacteres. They were grouped with Bacteroidetes and Deltaproteobacteria, respectively, their nearest neighbors^80,81^ shown in the tree.

Eukaryotic and archaeal NusG homologs were obtained by running 3 rounds of PSI-BLAST on the nr database with the following seed sequences: L1IE32, A0A0N95N5M7, UPI0005F5777A, A0A2E6HKN0. Redundant sequences were removed using CD-HIT^82^ at a 98% sequence identity threshold (at least 1 amino acid difference).

### Figures

Figures 1c, 2a, 2b, 3a, 3b, 4a, b were generated using Matplotlib^83^. The figures of all protein structures (Figs. 1a, 3c) were generated using PyMOL^84^. Fig. S1 was generated with seaborn^85^.

### Reporting summary

Further information on research design is available in the Nature Research Reporting Summary linked to this article.

## Supplementary information

Supplementary Information

Description of Additional Supplementary Files

Supplementary Data 1

Supplementary Data 2

Reporting summary

## Data Availability

Tabular data are provided in the Source Data File. Data recording all predictions are included in Supplementary Data 1 (bacteria and some archaea and eukaryotes) and Supplementary Data 2 (expanded predictions for archea and eukaryotes). Additional data are available at https://github.com/ncbi/sequence_space. Chemical shift assignments were deposited in the Biomolecular Magnetic Resonance Bank (BMRB) with the following accession codes: 51429 [10.13018/BMR51429] (Full-length Variant 5 (CTD only)), 51428 [10.13018/BMR51428] (Variant 5 isolated CTD), 51433 [10.13018/BMR51433] (Variant 8 CTD). PDB accession codes used in Fig. 1: 6ZTJ [10.2210/pdb6ZTJ/pdb] (*E. coli* 70S-RNAP expressome. Complex in NusG-coupled state, 38 nt intervening mRNA, chain CF), 5OND [10.2210/pdb5OND/pdb] (RfaH from *Escherichia coli* in complex with *ops* DNA, chain A), and 6C6S [10.2210/pdb6C6S/pdb] (CryoEM structure of the *E. coli* RNA polymerase elongation complex bound with RfaH, chain D). PDB accession codes used in Fig. 3: 5OND [10.2210/pdb5OND/pdb] (RfaH from *Escherichia coli* in complex with *ops* DNA, chain A), 2LCL [10.2210/pdb2LCL/pdb] (Solution Structure of RfaH carboxyterminal domain, chain A). Constructs for protein expression are available upon request. Source data are provided with this paper.

## Source data

Source Data

## Code availability

Code used to generate the predictions reported in this manuscript can be found at: https://github.com/ncbi/sequence_space.
