# Supplementary material for: Many dissimilar NusG protein domains switch between α-helix and β-sheet folds
Source: Nat Commun. 2022 Jul 1;13:3802. doi: 10.1038/s41467-022-31532-9 (PMC9247905; doi:10.1038/s41467-022-31532-9)
Supplement: Supplementary file 1 — Supplementary Information [file 41467_2022_31532_MOESM1_ESM.pdf]

Supplementary Information:

Many dissimilar NusG protein domains switch between α-helix and β-sheet folds

L. L. Porter *et al.*

Supplementary Figure 1

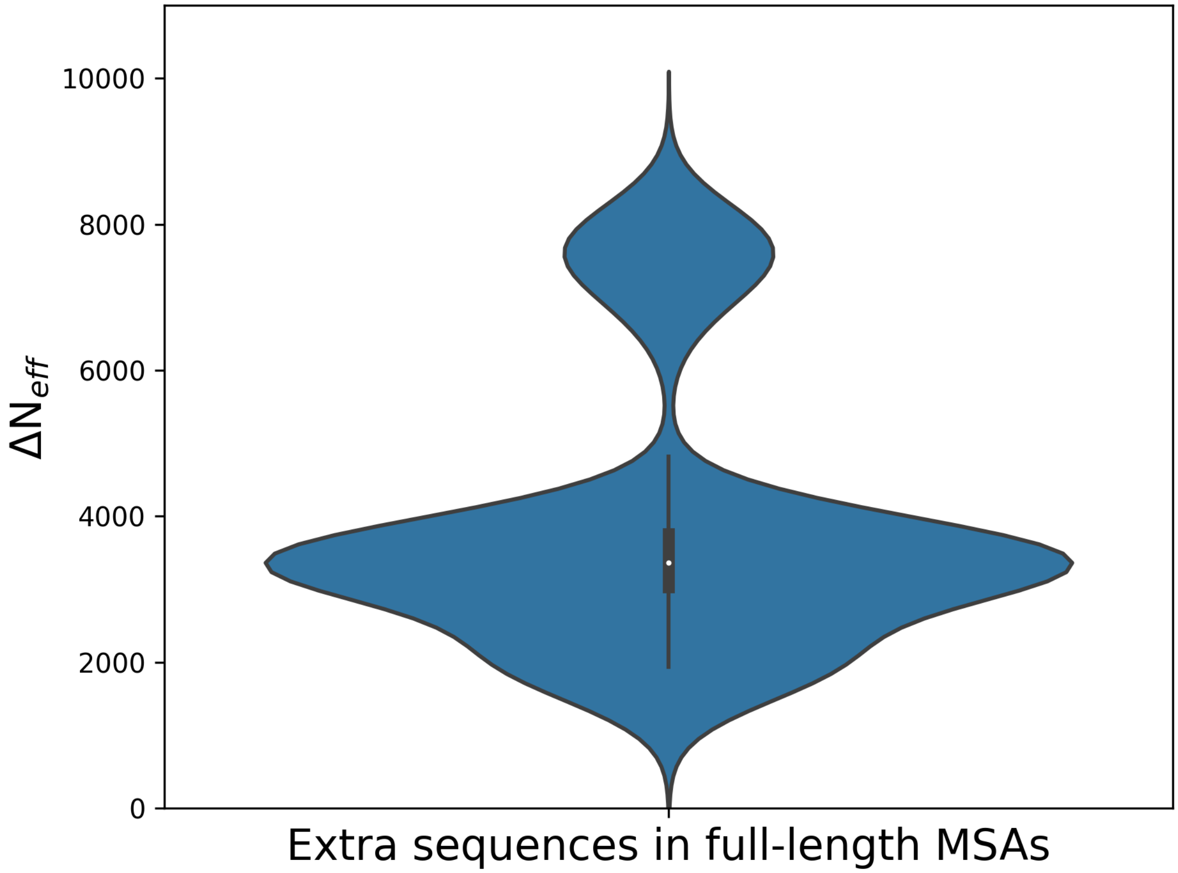

Violin plot of MSA depth differences between full-length NusG and $NusG^{SP}$ sequences and their corresponding CTD sequences. Mean/median full-length MSAs are effectively 3842/3368 sequences deeper than those from their corresponding CTDs. MSA depths were calculated as N_{eff}[1] (Methods). Bold black line spans upper and lower quartiles, thinner black line spans upper/lower extremes; white dot corresponds to median ΔN_{eff} value (3368).

Supplementary Figure 2

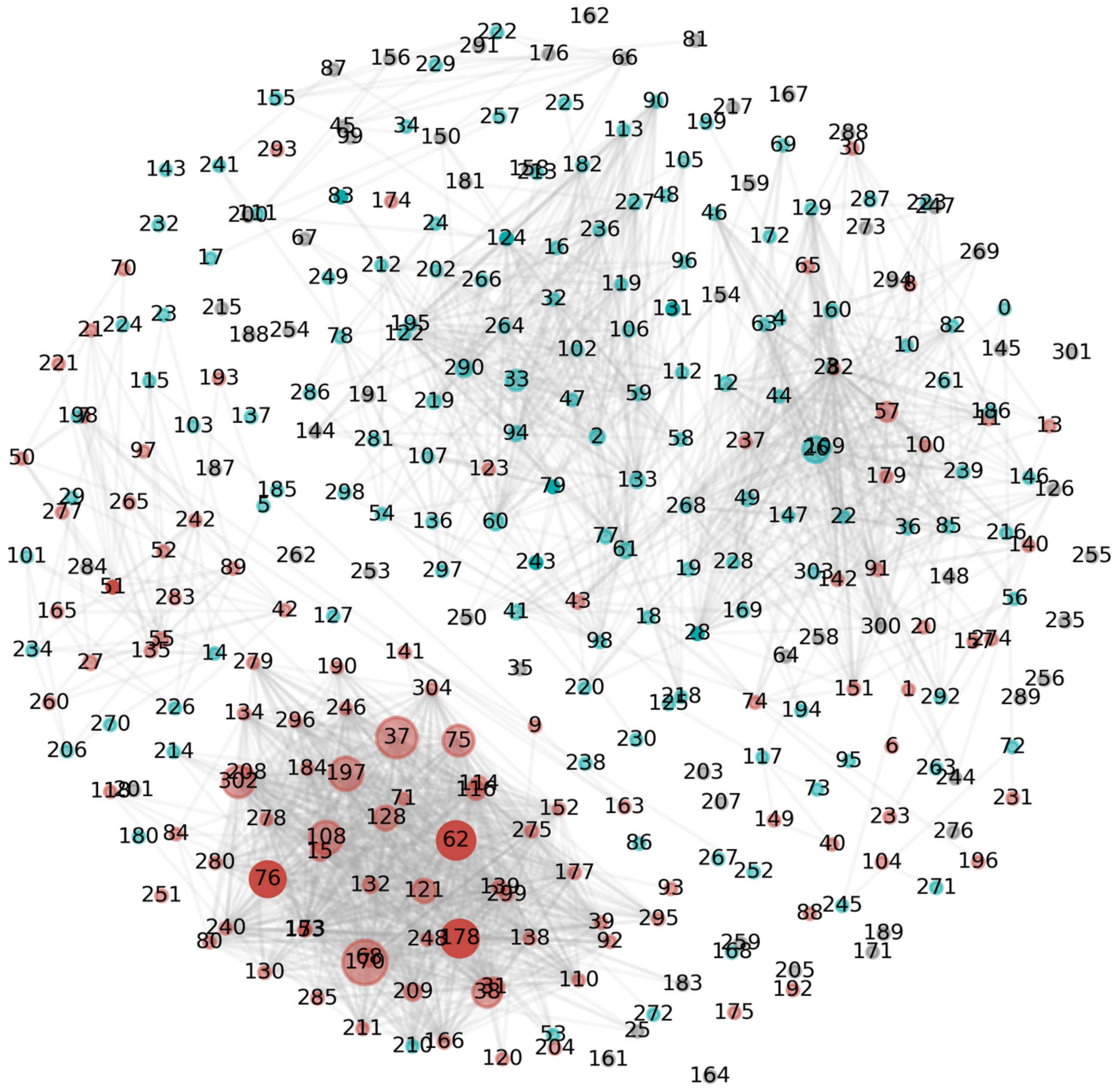

Sequence space diagram with cluster numbers labeled. Numbers correspond to the Cluster IDs (column 3) in Supplementary Data 1.

Supplementary Figure 3

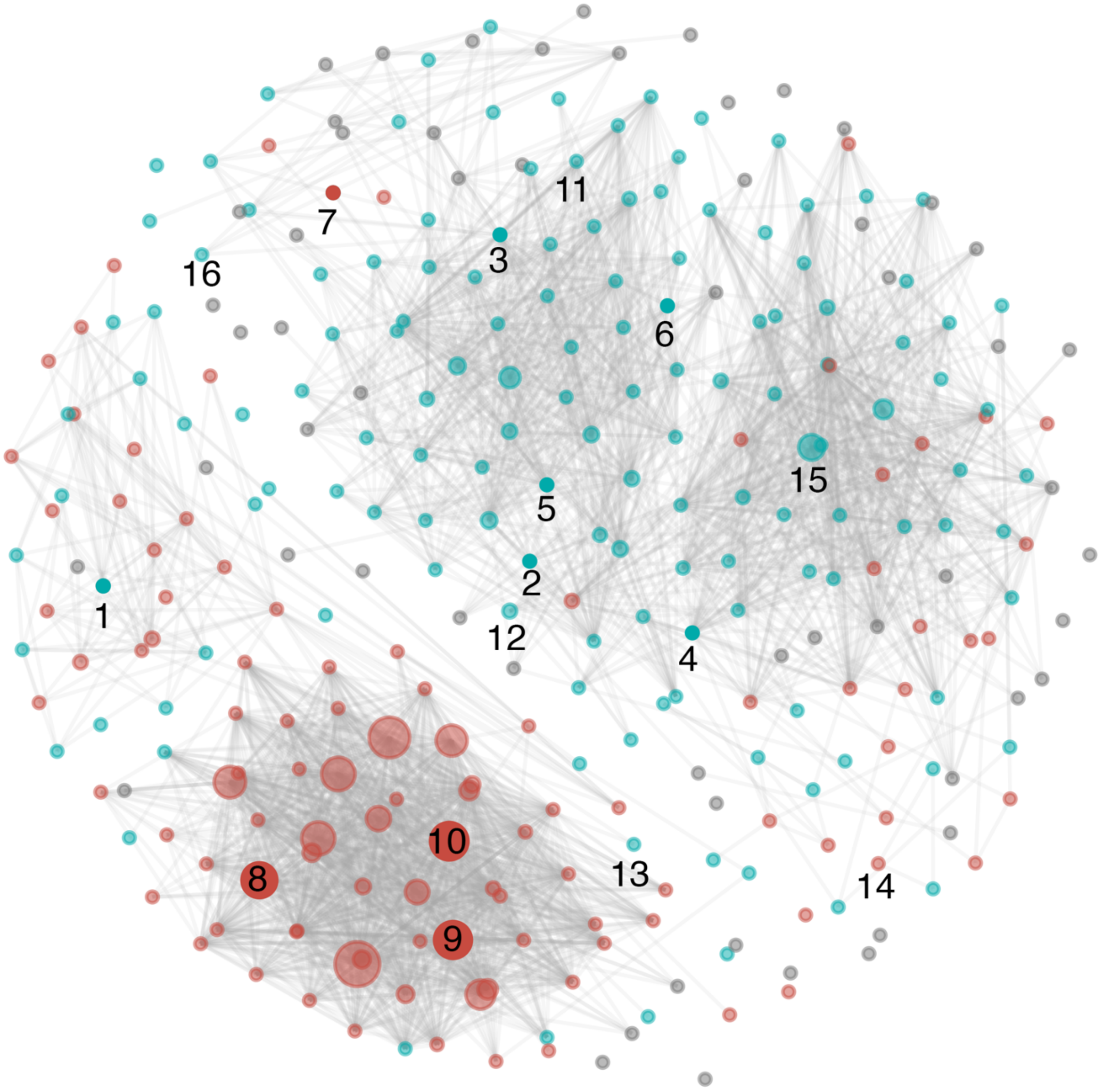

All sequence space constructs tested. Constructs 1-10 are labeled as in Fig. 2. Constructs 11-16 were tested, but CD spectra could not be obtained because they did not express (Constructs 11-13 and 15-16) or because they were insoluble (Construct 14). With the exceptions of Constructs 8-10, all labels are directly below the nodes from which sequences were selected. Teal/red nodes: predicted to/not to switch folds on average; no high-confidence predictions were made for gray nodes. More information about each construct can be found in Supplementary Table 1. Nodes 1 and 7 were colored differently from their average predictions (single folding, Node 1; fold-switching, Node 7) to highlight the prediction of the sequence validated experimentally, which differed from the average.

Supplementary Figure 4

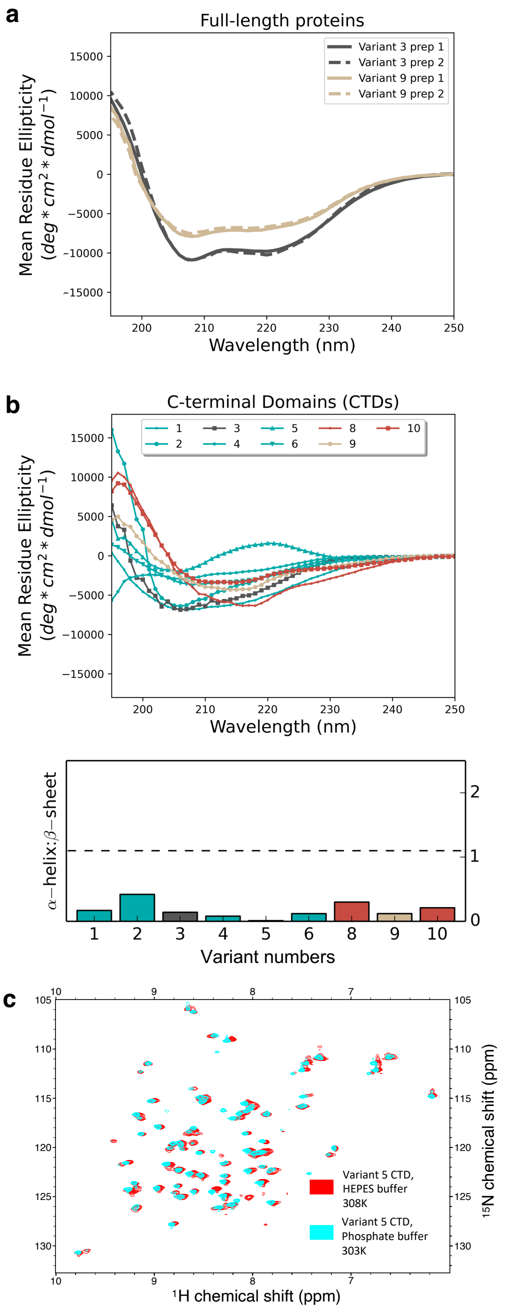

(a) Far-UV circular dichroism (CD) spectra of two different *E. coli* RfaH preps differ significantly from two different *E. coli* NusG preps. By contrast, CD spectra of the two different preps of both RfaH and NusG are nearly identical to one another. (b) Far-UV CD spectra of 9 CTDs fold predominantly into β-sheets. Spectra are shown above and estimated secondary structures shown below. Reference spectra of *E. coli* RfaH (Variant 3) and *E. coli* NusG (Variant 9) are colored gray and beige, respectively. All variants were estimated to have 27.3% (2)-43.0% (3) β-strand content, while α-helical content ranged from 0.0% (4 and 5)-8.4% (2). Secondary structure content was estimated by the BestSel server[2]. Variant numbers correspond to those in Fig. 2. Dotted line represents the helix:strand ratio of *E. coli* RfaH (variant 3), whose structure is known and whose CD spectrum has the lowest helical content of all 6 variants. (c) Variant 5 CTD assumes the same fold under different buffer conditions and temperatures. The 2D ^{1}H-^{15}N HSQCs of Variant 5 CTD under different conditions are nearly superimposable. Conditions from Fig. 2c (red, 25 mM HEPES, 50 mM NaCl, 5% glycerol, pH 7.5, T=308K) overlays with the 2D ^{1}H-^{15}N HSQC of Variant 5 CTD, whose resonances were assigned (cyan, 100 mM potassium phosphate, pH 7.4, T=303K). Source data are provided as a Source Data file.

Supplementary Figure 5

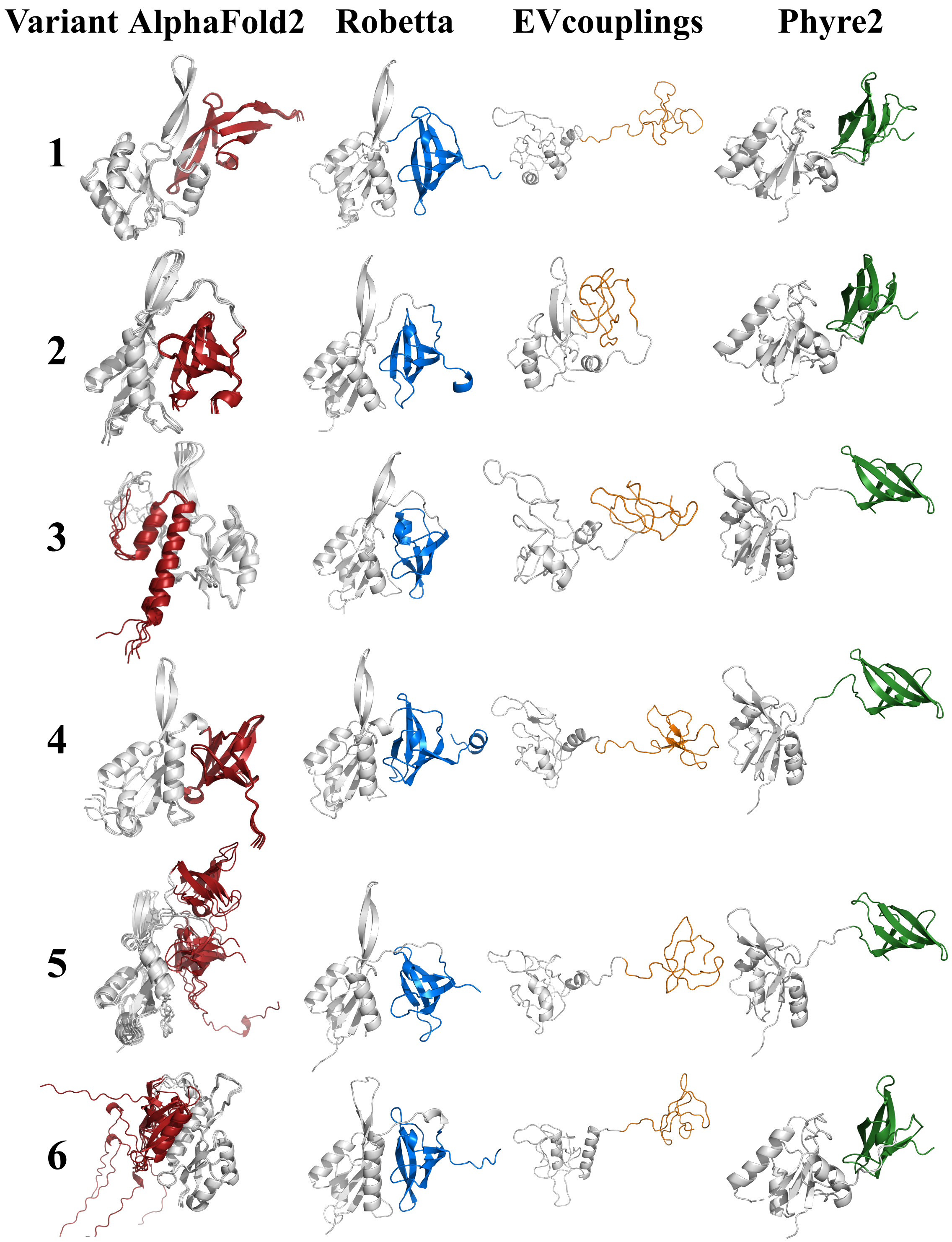

CTDs of the lowest-energy models for 6 proteins with RfaH-like folds (helical hairpin) are predicted assume β-sheet folds, including *E. coli* RfaH (Construct 3), which has an experimentally validated structure. CTDs are colored burgundy (AlphaFold2), blue (Robetta), orange (EVcouplings), green (Phyre2). Variant numbers correspond to those in Fig. 2. Top 5 AlphaFold2 models are homogeneous enough to be shown; other models are not.

Supplementary Figure 6

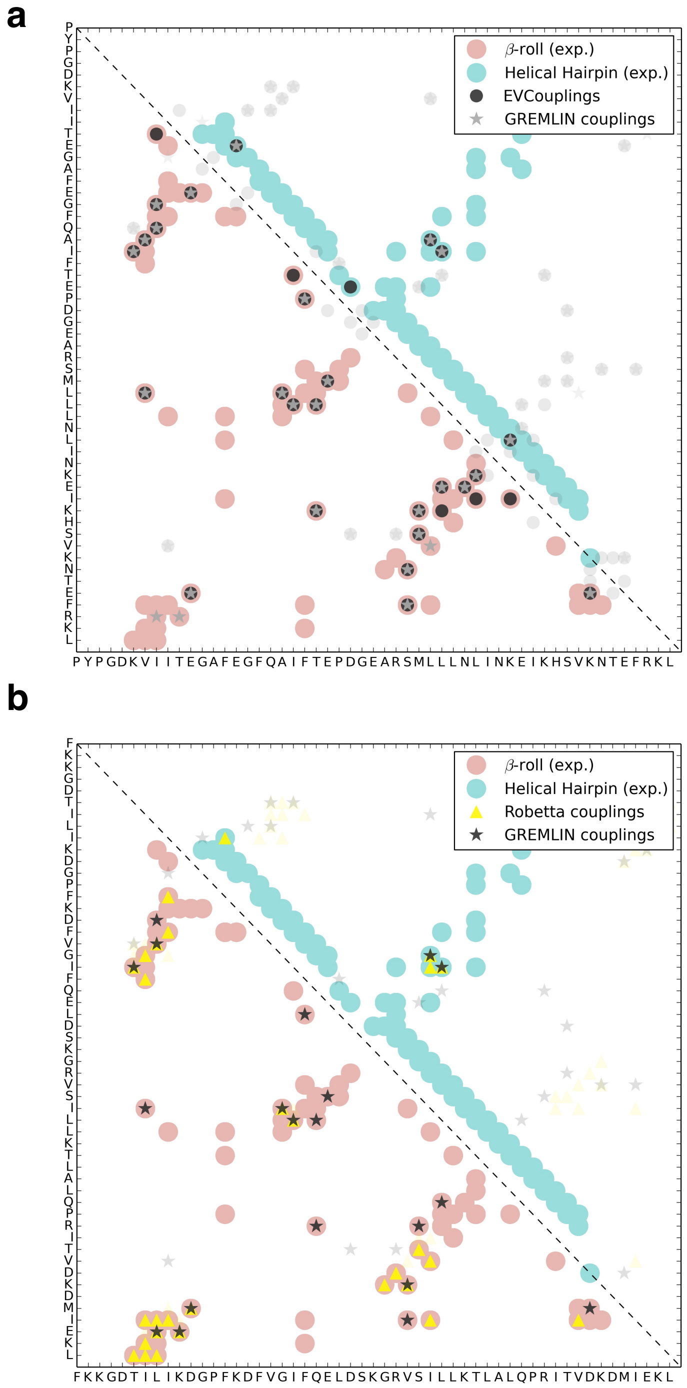

GREMLIN couplings calculated from EVCouplings (a) and Robetta (b) sequence alignments largely match contacts from the experimentally determined β-roll fold (red, PDB ID 2LCL) but did not match any contacts unique to the helical hairpin fold (teal, PDB ID 5OND_A).
Source data are provided as a Source Data file.

Supplementary Figure 7

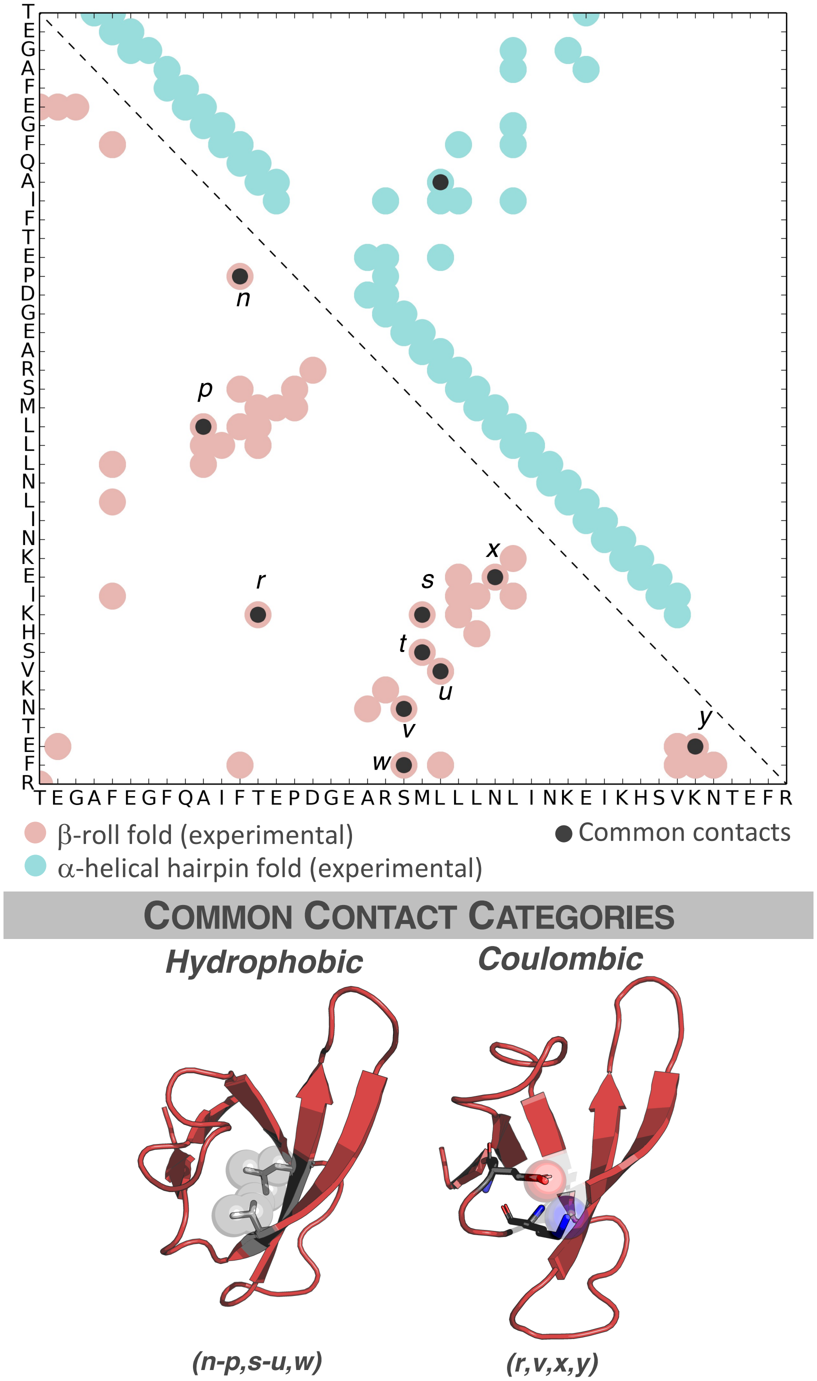

The single-fold paradigm biases protein structure predictions. EVCouplings and Robetta identify conserved residue-residue contacts (gray circles) corresponding to the β-roll fold of *E. coli* RfaH (PDB ID: 2LCL_A, red circles) but not the α-helical hairpin fold (PDB ID: 5OND_A, teal circles). Italicized letters correspond to individual contacts observed in the β-roll fold. The only helical contact identified is also a contact in the β-roll fold (contact *q*); its helical form is contact *i* in Fig. 3. Contact categories and their corresponding letters are shown below.
Source data are provided as a Source Data file.

Supplementary Figure 8

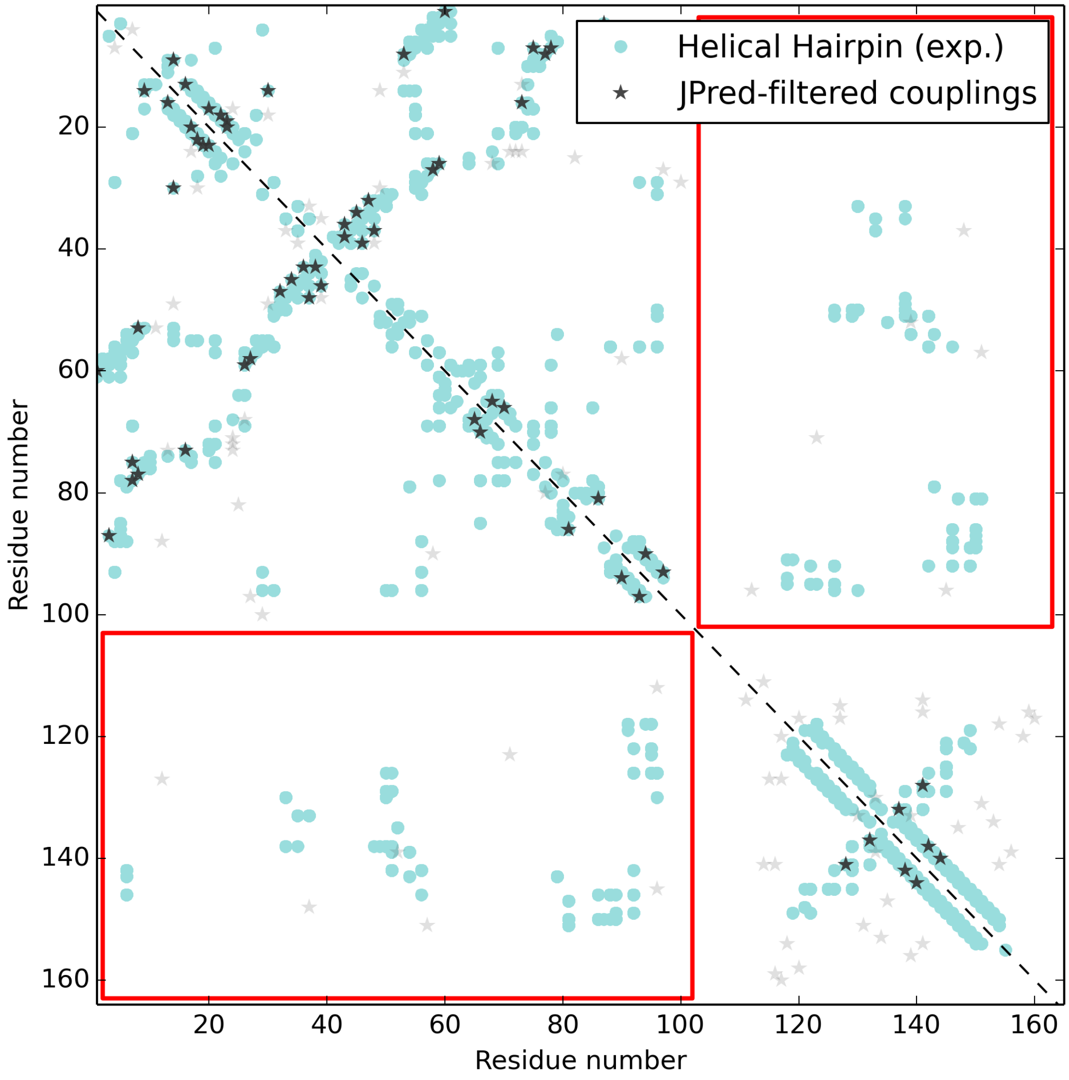

JPred-filtered couplings of putative full-length fold switchers calculated by GREMLIN. No interdomain contacts (found within red boxes) were consistent with the experimentally determined structure of full-length RfaH (PDB ID: 5OND_A). Source data are provided as a Source Data file.

Supplementary Table 1. Sequences of all variants tested (see also Supplementary Figure 3).

#	ID	Cluster	Annotation	Phylum/Class	Pred	%H<->E	Express?	Soluble?
1	UPI000E4E22B5	51	LoaP	Firmicutes	FS	9%	Y	Y
2	A0A0S4NBF0	243	RfaH	Candidatus Kryptonia	FS	53%	Y	Y
3	Q0TAL4	124	RfaH	Gammaproteobacteria	FS	44%	Y	Y
4	B3EDK9	28	NusG	Chlorobi	FS	7%	Y	Y
5	A0A2J6WKD6	79	NGN domain-containing protein	Deferribacteres	FS	81%	Y	Y
6	E8N6B2	131	Putative RfaH	Chloroflexi	FS	50%	Y	Y
7	Q9F769	83	UpbY	Bacteroidetes	NFS	2%	Y	Y
8	CUU00518.1 (A0A0P1LTF1)	76	NusG	Candidatus Kryptonia	NFS	0%	Y	Y
9	P0AFG0 (A0A077QHU1)	178	NusG	Gammaproteobacteria	NFS	0%	Y	Y
10	A0A1W1XWG8	62	NusG	Deltaproteobacteria	NFS	0%	Y	Y
11	*A1VS04*	*105*	*NusG*	*Betaproteobacteria*	*FS*	*65%*	*N*	-
12	*A0A1W1XVU0*	*41*	*NusG*	*Deltaproteobacteria*	*NFS*	*0%*	*N*	-
13	*A0A348AQW0*	*86*	*RfaH*	*Firmicutes*	*FS*	*30%*	*N*	-
14	*Q984H9*	*40*	*Mlr7998*	*Alphaproteobacteria*	*FS*	*38%*	*Y*	*N*
15	*A0A1M6KQH4*	*26*	*NusG*	*Bacteroidetes*	*FS*	*37%*	*N*	-
16	*A0A0F6QDM6*	*17*	*RfaH (on actX gene)*	*Gammaproteobacteria*	*FS*	*57%*	*N*	-

Supplementary Table 2. Sequences of all variants successfully purified and characterized by circular dichroism. FS indicates predicted fold switcher: Y means Yes, N means No.

Variant	FS	Sequence
1	Y	MMKPWYVLYVMGGKEQKILSLLNKGEDIKAFTPWKEVMHRVQGKRILVKKPLFPSYVFLE<br>TELDPAVFHQKLMLYKSQINGILKELKYEDDISALHTEERAYLEGLMDEEHNVRLSKGEI<br>LDGEVIITEGPLKGYESNIIRIDRHKRRAILNVRMNNQDLQVDVSLEIVKKIESQK
2	Y	MDLNWYVLQTKPKQENLVESYLNLANIEVFNPKIQEIRYIGEKRKKITVLLFPCYVFAKL<br>NPSLFDLVIYTRGVRKILGVNGRPKPIKESIIETIKERIRENSYIYVPENYEEFQLCQGD<br>YVVVVDGPLKGFAGIVERINGSKAIVMLISMDYQVKADIPKFLLRKVDPEILE
3	Y	MQSWYLLYCKRGQLQRAQEHLERQAVNCLAPMITLEKIVRGKRTAVSEPLFPNYLFVEFDP<br>EVIHTTTINATRGVSHFVRFGASPAIVPSAVIHQLSVYKPKDIVDPATPYPGDKVIITEGA<br>FEGFQAIFTEPDGEARSMLLLNLINKEIKHSVKNTEFRKL
4	Y	MKVTDRNSCWYAVYVRSRYEKKVHRMFLEKEVEAFLPLLETWRQWSDRKKKVSEPLFRGY<br>VFVNIDMKAEHIKVLDTDGVVKFIGIGKTPSVISSRDIDWIKKLVREPDAVRRIVASLPP<br>GQKVMVTAGPFKGLEGVVVKEGRESRLVVYFDRIMQGIEVSIYPELLSPIHAVGTEEQNE<br>TGFY
5	Y	MESFLNWYLIYTKVKKEDYLEQLLTEAGLEVLNPKIKKTKTVRNKKKEVIDPLFPCYLFV<br>KADLNVHLRIISYTQGIRRLVGGSNPTIVPIEIIDTIKSRMVDGFIDTKSEEFKKGDTIL<br>IKDGPFKDFVGIFQEELDSKGRVSILLKTLALQPRITVDKDMIEKLHN
6	Y	MSKKWYAIQSKPNKEQALCEQFQSRGIEVFYPQIRVNPVNPRARKIRPYFPGYLFVHVDL<br>DEVGLSVIRWIPFARGVVSFSNEPASVPDNLIEAIRRRVDEVNRAGGELLETLKPGEPVL<br>IQEGPFAGYEAIFDVRLSGKERVRVLIQLLSQRYIPVEMQVGSLKPLKTKNKDKPHPL
7	N	MSEQQKYWFAARTRDKQEFAIRDSLEKLKTELDLNYYLPTQFVIRQLKYRRKRVEVPVIK<br>NLIFIQATKQDACDISNKYNIQLFYMKDLLTRAMLIVPDKQMQDFIFVMDLDPNGVSFDN<br>DHLSVGSRVQVVKGDFCGVEGELASEANKTYVVIRIAGVLSASVKVPKSYLRVI
8	N	MARRWYAVRTYSGHENRVKKFIENEIAEGKFKDKIFNVLVPTEKVTVVREGRKKSRVKAF<br>FPGYILIEAEMDDEVKNFIRAVPSVVSFVGPKGNPVPLREDEVERFIGKPEGAELERIDV<br>PFRVGDSVKVIDGPFTDFSGVVQEVNSEKMKLKVMINIFGRKTPVELDFTQVEIEK
9	N	MSEAPKKRWYVVQAFSGFEGRVATSLREHIKLHNMEDLFGEVMVPTEEVVEIRGGQRRKS<br>ERKFFPGYVLVQMVMNDASWHLVRSVPRVMGFIGGTSDRPAPISDKEVDAIMNRLQQVGD<br>KPRPKTLFEPGEMVRVNDGPFADFNGVVEEVDYEKSRLKVSVSIFGRATPVELDFSQVEK<br>A
10	N	MRMDEGLSRSGGDRVAKQWYIVHTYSGFEHRVKAALQERIKAAGKEEYFGQILVPTEKVV<br>ELVKGERKSSSRKFYPGYIVVEMELNDETWHLVRHTPKVTGFIGSQERPIPLSEEEANAI<br>IQQMEEGIQKPRPKYQFEKGEEVRVVDGPFASFNGVVEQVIPEKGKVRVLVTIFGRSTPV<br>ELDFVQIQRL

Supplementary Table 3. Sequences of CTDs whose CD spectra were collected. See also Supplementary Fig. 4. These are also the sequences of the Variant 5 and 8 CTDs used for NMR. FS indicates predicted fold switcher: Y means Yes, N means No.

CTD Variant	FS	Sequence
1	Y	TSLSKGEILDGEVIITEGPLKGYESNIIRIDRHKRRAILNVRMNNQDLQVDVSLEIVKKIESQK
2	Y	TSWIKERIRENSYIYVPENYEEFQLCQGDYVVVVDGPLKGFAGIVERINGSKAIVMLISMDYQV<br>KADIPKFLLRKVDPEILE
3	Y	LSVYPKDIVDPATPYPGDKVIITEGAFEGFQAIFTEPDGEARSMLLLNLINKEIKHSVKNTEFR<br>KL
4	Y	TSRIVASLPPGQKVMVTAGPFKGLEGVVVKEGRESRLVVYFDRIMQGIEVSIYPELLSPIHAVG<br>TEEQNETGFY
5	Y	MVDGFIDTKSEEFKKGDTILIKDGPFKDFVGIFQEELDSKGRVSILLKTL<br>ALQPRITVDKDMIEKLHN
6	Y	TSWELLETLKPGEPVLIQEGPFAGYEAIFDVRLSGKERVRVLIQLLSQRYIPVEMQVGSLKPLK<br>TKNKDKPHPL
7	N	TSWFDNDHLSVGSRVQVVKGDFCGVEGELASEANKTYVVIRIAGVLSASVKVPKSYLRVI
8	N	AELERIDVPFRVGDSVKVIDGPFTDFSGVVQEVNSEKMKLKVMINIFGRK<br>TPVELDFTQVEIEK
9	N	TSWRPKTLFEPGEMVRVNDGPFADFNGVVEEVDYEKSRLKVSVSIFGRATPVELDFSQVEKA
10	N	TSRPKYQFEKGEEVRVVDGPFASFNGVVEQVIPEKGKVRVLVTIFGRSTPVELDFVQIQRL

Supplementary Table 4. Oligonucleotide sequences used to make C-terminal domain constructs.

Primer Name	Oligonucleotide sequence
Variant 1 Forward	AGCCTGAGCAAAGGTGAAATTC
Variant 1 Reverse	AGTCAAAGCTTTGAAGAGCTTG
Variant 2 Forward	CTGGATCAAAGAGCGCATTCG
Variant 2 Reverse	CTAGTCAAAGCTTTGAAGAGCTTG
Variant 3 Forward	CCGAAGGATATTGTTGATCCGG
Variant 3 Reverse	CATCAAAGCTTTGAAGAGCTTGTC
Variant 4 Forward	ACCAGCCGTATTGTTGCAAGCCTG
Variant 4 Reverse	CAAAGCTTTGAAGAGCTTG
Variant 5 Forward	GTGGATGGTTTTATCGATACC
Variant 5 Reverse	CATCAAAGCTTTGAAGAGCTTGTC
Variant 6 Forward	CTGGGAACTGCTGGAAACCCTG
Variant 6 Reverse	CTGGTCAAAGCTTTGAAGAGCTTG
Variant 7 Forward	CTGGTTTGATAATGATCATCTGAGC
Variant 7 Reverse	CTGGTCAAAGCTTTGAAGAGCTTG
Variant 8 Forward	GCAGAACTCGAACGTATTG
Variant 8 Reverse	CAAAGCTTTGAAGAGCTT
Variant 9 Forward	CTGGCGTCCGAAAACACTGTTTG
Variant 9 Reverse	CTCGTCAAAGCTTTGAAGAGCTTG
Variant 10 Forward	ACCAGCCGTCCGAAATATCAGTTTG
Variant 10 Reverse	CAAAGCTTTGAAGAGCTTG

Supplementary References.

1 Wu, T., Hou, J., Adhikari, B. & Cheng, J. Analysis of several key factors influencing deep learning-based inter-residue contact prediction. *Bioinformatics* **36**, 1091-1098, doi:10.1093/bioinformatics/btz679 (2020).

2 Micsonai, A. *et al.* BeStSel: webserver for secondary structure and fold prediction for protein CD spectroscopy. *Nucleic Acids Res*, doi:10.1093/nar/gkac345 (2022).
