# Supplementary material for: Many dissimilar NusG protein domains switch between α-helix and β-sheet folds
Source: Nat Commun. 2022 Jul 1;13:3802. doi: 10.1038/s41467-022-31532-9 (PMC9247905; doi:10.1038/s41467-022-31532-9)
Supplement: Supplementary file 2 — Description of Additional Supplementary Files [file 41467_2022_31532_MOESM2_ESM.docx]

Filename: Supplementary Data 1.

Description: Annotations and predictions of all sequences identified in the NusG superfamily (Attached Separately). Overall Pred: overall prediction, NFS: not fold-switching; FS: fold switching. Overall AtoB Disc: Average α-helix <-> β-strand discrepancies between the two predictions and their reference β-sheet predictions. Confidences: Good confidence: at least 5 sequences hit; Low confidence: fewer than 5 sequences hit. Preds used: 1: JPred database only; 2: Uniref Databse only, 3: predictions from both databases. Both databases were used when both confidences were good; otherwise, only the database with good confidence were used. If neither database yielded high-confidence sequence hits, no prediction was made. %identity: percent identity of original sequence from UniRef90 to Ensemble sequence. Minimum identity was 90%. *secE* start/stop and *rplK* start/stop indicate proximity to genes in the housekeeping NusG operon.

Filename: Supplementary Data 2.

Description: Additional annotations and predictions of archaeal and eukaryotic sequences used to determine the tree in Fig. 4. Overall Pred: overall prediction, NFS: not fold-switching; FS: fold switching. Overall AtoB Disc: Average α-helix <-> β-strand discrepancies between the two predictions and their reference β-sheet predictions. Confidences: Good confidence: at least 5 sequences hit; Low confidence: fewer than 5 sequences hit. Preds used: 1: JPred database only; 2: Uniref Databse only, 3: predictions from both databases. Both databases were used when both confidences were good; otherwise, only the database with good confidence were used. If neither database yielded high-confidence sequence hits, no prediction was made. (Attached separately.)
